# Thrifty-Eating Behavior Phenotype at the Food Court – Programming Goes Beyond Food Preferences

**DOI:** 10.3389/fendo.2022.882532

**Published:** 2022-05-23

**Authors:** Roberta Dalle Molle, Euclides José de Mendonça Filho, Luciano Minuzzi, Tania Diniz Machado, Roberta Sena Reis, Danitsa Marcos Rodrigues, Amanda Brondani Mucellini, Alexandre Rosa Franco, Augusto Buchweitz, Rudineia Toazza, Andressa Bortoluzzi, Giovanni Abrahão Salum, Sonia Boscenco, Michael J. Meaney, Robert D. Levitan, Gisele Gus Manfro, Patricia Pelufo Silveira

**Affiliations:** ^1^Programa de Pós-Graduação em Ciências da Nutrição, Universidade Federal de Ciências da Saúde de Porto Alegre, Porto Alegre, Brazil; ^2^Ludmer Centre for Neuroinformatics and Mental Health, Douglas Hospital Research Center, Montreal, QC, Canada; ^3^Programa de Pós-Graduação em Saúde da Criança e do Adolescente, Faculdade de Medicina, Hospital de Clínicas de Porto Alegre, Universidade Federal do Rio Grande do Sul, Porto Alegre, Brazil; ^4^Department of Psychiatry, McGill University, Montreal, QC, Canada; ^5^Department of Psychiatry and Behavioural Neurosciences, McMaster University, Hamilton, ON, Canada; ^6^Faculdade de Nutrição, Universidade Federal de Goiás, Goiânia, Brazil; ^7^Programa de Pós-Graduação em Neurociências, Instituto de Ciências Básicas da Saúde, Universidade Federal do Rio Grande do Sul, Porto Alegre, Brazil; ^8^Programa de Pós-Graduação em Ciências Médicas: Psiquiatria, Faculdade de Medicina, Hospital de Clínicas de Porto Alegre, Universidade Federal do Rio Grande do Sul, Porto Alegre, Brazil; ^9^Instituto do Cérebro (InsCer), Pontifícia Universidade Católica do Rio Grande do Sul (PUCRS), Porto Alegre, Brazil; ^10^Faculdade de Medicina, Programa de Pós-Graduação em Medicina e Ciências da Saúde, Pontifícia Universidade Católica do Rio Grande do Sul (PUCRS), Porto Alegre, Brazil; ^11^Faculdade de Engenharia, Programa de Pós-Graduação em Engenharia Elétrica, PUCRS, Porto Alegre, Brazil; ^12^Faculdade de Letras, Programa de Pós-Graduação em Letras, Linguística, Pontifícia Universidade Católica do Rio Grande do Sul (PUCRS), Porto Alegre, Brazil; ^13^Singapore Institute for Clinical Sciences, Agency for Science, Technology and Research (A∗STAR), Singapore, Singapore; ^14^Department of Psychiatry, University of Toronto and Centre for Addiction and Mental Health, Toronto, ON, Canada

**Keywords:** small for gestational age (SGA), feeding behavior, resting state fMRI, functional connectivity, orbitofrontal cortex

## Abstract

**Introduction:**

Prenatal growth impairment leads to higher preference for palatable foods in comparison to normal prenatal growth subjects, which can contribute to increased body fat mass and a higher risk for developing chronic diseases in small-for-gestational-age (SGA) individuals throughout life. This study aimed to investigate the effect of SGA on feeding behavior in children and adolescents, as well as resting-state connectivity between areas related to reward, self-control, and value determination, such as orbitofrontal cortex (OFC), dorsolateral prefrontal cortex (DL-PFC), amygdala and dorsal striatum (DS).

**Methods:**

Caregivers and their offspring were recruited from two independent cohorts in Brazil (PROTAIA) and Canada (MAVAN). Both cohorts included anthropometric measurements, food choice tasks, and resting-state functional magnetic resonance imaging (fMRI) data.

**Results:**

In the Brazilian sample (17 ± 0.28 years, n=70), 21.4% of adolescents were classified as SGA. They exhibited lower monetary-related expenditure to buy a snack compared to controls in the food choice test. Decreased functional connectivity (n=40) between left OFC and left DL-PFC; and between right OFC and: left amygdala, right DS, and left DS were observed in the Brazilian SGA participants. Canadian SGA participants (14.9%) had non-significant differences in comparison with controls in a food choice task at 4 years old ( ± 0.01, n=315). At a follow-up brain scan visit (10.21 ± 0.140 years, n=49), SGA participants (28.6%) exhibited higher connectivity between the left OFC and left DL-PFC, also higher connectivity between the left OFC and right DL-PFC. We did not observe significant anthropometric neither nutrients’ intake differences between groups in both samples.

**Conclusions:**

Resting-state fMRI results showed that SGA individuals had altered connectivity between areas involved in encoding the subjective value for available goods and decision-making in both samples, which can pose them in disadvantage when facing food options daily. Over the years, the cumulative exposure to particular food cues together with the altered behavior towards food, such as food purchasing, as seen in the adolescent cohort, can play a role in the long-term risk for developing chronic non-communicable diseases.

## 1 Introduction

According to the Developmental Origins of Health and Disease (DOHaD) framework, maternal conditions and environmental cues during pregnancy may impact health and disease in adulthood. Adversities experienced during embryonic and fetal development may program the offspring for increased predisposition to non-communicable chronic diseases (1). The most common outcome that reflects fetal adversity is birth weight. A recent meta-analysis found associations between being born with low birth weight or small for gestational age (SGA) and incidence of cardiometabolic disease, glucidic metabolism disorders, and metabolic syndrome (2).

In developing countries, poor gestational nutrition and low pre-pregnancy weight are the main determinants of intrauterine growth restriction (IUGR), whereas in developed countries the main risk factor is cigarette smoking (3). DOHaD emerged from epidemiological studies conducted in developed countries (4), however in developing countries the association between fetal adversity and the development of non-communicable chronic diseases has also been observed (5). This suggests that independently of the cause of insufficient fetal growth, scarcity of nutrients activates a process of physiological adaptation in the fetus to guarantee survival (6). The literature of underlying mechanisms of prenatal programming is still scarce but may include prenatal structural defects, metabolic (mitochondrial dysfunction, oxidative stress, protein modification), epigenetic and glucocorticoid signaling-related mechanisms (1).

A body of clinical and experimental evidence shows that the exposure to intrauterine adverse events, often culminating with IUGR, alters the individuals’ feeding preferences and eating behaviors, increasing their intake of foods rich in carbohydrates and/or fat (7). The increased preference for palatable foods may lead to subtle but persistent nutritional imbalances and contribute to the development of adult chronic non-communicable diseases in individuals who did not grow as much as expected in the uterus. Awareness and deep comprehension about these behavioral peculiarities and their neuropsychological basis may open an important venue for disease prevention in these subjects. Therefore, childhood and adolescence are important life stages characterized by sensitive periods of development that need further investigation, since the cumulative effects of such nutritional imbalances might not be established yet.

Eating behavior is regulated by three interconnected central hubs: the hypothalamus, involved in the homeostatic control; the “appetitive network”, which consists of four regions (insula, medial temporal lobe, OFC/vmPFC, and striatum) responsible to encode the current incentive salience of sensed and available food rewards; and the ventromedial, dorsomedial, and dorsolateral prefrontal cortices, which control goal-oriented and self-regulated behavior (8). The OFC plays a critical role in the choice evaluation; it encodes the value of goals in decision-making (9), and is more frequently activated to current energy balance information in response to food cues (10). According to Suzuki et al. (11), the information about the nutritive attributes of foods is represented in the lateral OFC, which integrates this information with the medial OFC to compute an overall value. Interestingly, obese individuals may have a relative inability to down-regulate the OFC response to high-calorie food cues following satiation (12) and after bariatric surgery OFC resting-state connectivity to mesolimbic areas is reduced (13).

This study was designed to contribute to the literature regarding fetal adversity programming of eating behavior throughout life by replicating the analyses in two independent cohorts. More specifically, we examined the effect of being born SGA on feeding behavior in children and adolescents, as well as on resting-state functional connectivity between areas related to reward, self-control, and value determination. Based on previous studies, we hypothesized that SGA individuals from the two sites would have: i) altered behavior towards food in different situations presented to them (e.g. when buying a snack or exposed to a buffet), in comparison to normal birth weight individuals; and ii) altered brain functional tonic connectivity between the OFC and brain regions involved in encoding the subjective value for available goods and decision-making.

## 2 Materials and Methods

### 2.1 Participants and Measurements

#### 2.1.1 PROTAIA

The adolescents and young adults selected for the study originated from a community sample selected from six schools that belong to the service area of the Santa Cecilia Health Unit, located in Porto Alegre, Rio Grande do Sul, Brazil. The students were invited to participate in the PROTAIA project (The multidimensional evaluation and treatment of anxiety in children and adolescents), which included psychiatric and nutritional assessment (14). Two-hundred-and-forty-two participants completed the first assessment; out of which 229 were eligible for the current study (six were excluded due to intellectual disability; seven due to kinship with another participant). The study design was to carry out a detailed reevaluation, five years later, in approximately 30% of the eligible sample, as the present study is the fourth wave of a long-term follow-up study [as suggested by Barbieri et al. (15)]. The reevaluation included: (1) psychiatric assessment; (2) anthropometric and feeding behavior assessment; and (3) functional neuroimaging scans. In the current study we focused on anthropometric, feeding behavior and brain resting-state connectivity data as the outcomes. Socioeconomic and psychiatric data were used to describe the sample. Data collection was performed in two laboratory visits both conducted during the morning. Seventy-five participants attended the first laboratory visit (behavioral data collection) in the Clinical Research Center of the Hospital de Clínicas de Porto Alegre (CPC - HCPA) and 43 attended the second laboratory visit, at the Brain Institute of Rio Grande do Sul - PUCRS (InsCer), when a functional magnetic resonance imaging (fMRI) protocol was performed.

The psychiatric evaluation was carried out using: (1) a structured clinical interview - Schedule for Affective Disorders and Schizophrenia for School-Age Children-Present and Lifetime Version (K-SADS-PL) (16), held with the adolescents and (2) the Brazilian version of the MINI instrument (International Neuropsychiatric Interview) (17), applied in the over-18s. These instruments were applied by medical and psychology students trained for such activity and supervised by a child/adolescent psychiatrist.

The socioeconomic classification was based on the ABEP score (Brazilian Association of Research Companies), which ranks the socioeconomic status according to the possession of certain items and the educational level of the head of the family: Class A – 35 to 46; Class B – 23 to 34; Class C - 14 to 22; Class D - 8 to 13 and Class E – 0 to 7. Maternal education was collected in years of schooling and classified using the cut-off point of eight years of education.

The study was approved by the Institutional Ethics Committee of the HCPA (GPPG/HCPA, project number 12-0254) and followed national and international guiding principles for research involving humans, including the Resolution 196/96 from the National Health Council. Ethics committee approval and a subsequent written informed consent were obtained from all participants prior to the study.

#### 2.1.2 MAVAN

Data from the Maternal Adversity, Vulnerability, and Neurodevelopment (MAVAN) prospective birth cohort (18) were used. Eligibility criteria for mothers included age 18 years old or older, singleton pregnancy, and fluency in French or English. Mothers were excluded from the study if they had severe chronic illness, placenta previa, a history of incompetent cervix, impending delivery, or had a fetus/infant born at gestational age <37 weeks or born with a major anomaly. Six hundred and thirty mother-child dyads were recruited during pregnancy in Montreal, QC and Hamilton, ON. They were assessed longitudinally, with multiple evaluations of both mother and child in-home and laboratory across the child’s development. In the current study, we focused on the feeding behavior assessment performed at 4 years old with 315 participants. Moreover, functional neuroimaging scans performed at the Montreal site at 10 years of age with 49 participants were analyzed. Socioeconomic data were used to describe the sample. The socioeconomic classification was based on household total gross income, being the cut-off point CAD 40,000 per year. Maternal education was classified according to the completion of high school.

The study was approved by the institutional review boards at hospitals and university affiliates: McGill University, l’Universite de Montreal, the Royal Victoria Hospital, Jewish General Hospital, Centre Hopitalier de l’Universite de Montreal, Hôpital Maisonneuve-Rosemont, St Joseph’s Hospital, and McMaster University. Informed consent was obtained from the parents/guardians of the participants.

### 2.2 Small for Gestational Age (SGA) Determination

Birth weight and gestational age data were collected from the child’s health record. The SGA classification was based on the birth weight ratio (BWR), which is the ratio between the infant birth weight and the sex-specific mean birth weight for each gestational age for the local population (19, 20). Participants were classified as SGA if they have a BWR of <0.85 (21).

### 2.3 Anthropometric Assessment

The anthropometric assessment was performed by trained researchers. Weight and height were measured using accurate and calibrated equipment (Filizola^®^, Harpenden^®^, TANITA^®^ and Perspective Enterprises^®^), and the participants were without shoes and in light clothing. The measures were performed in duplicate, and the average value was adopted. Body mass index (BMI) was calculated as weight in kilograms divided by height in meters squared (kg/m^2^). Z-scores for BMI were calculated according to World Health Organization (WHO) standards (22). Children from 0 to 5 years of age with BMI-for-age Z-scores above +2 were classified as overweight and +3 as obese. Children above 5 years old and adolescents were classified as overweight when the BMI-for-age Z-scores were above +1 and as obese when above +2 (23).

### 2.4 Feeding Behavior Assessment

#### 2.4.1 PROTAIA

Anthropometric measures were performed in the morning while participants were fasting. Afterwards, they received a voucher to purchase snacks of their choice in the cafeteria of the HCPA Clinical Research Center (CPC-HCPA); the voucher offered had the same monetary value for all participants, and was sufficient to purchase different items (e.g. the voucher would be enough to purchase a sandwich plus a small soft drink or coffee, plus a small desert) (**Supplementary Figure S1**). Participants were free to choose any snack on the cafeteria, as long as it did not cost more than the voucher. If the participant did not use the full value of the voucher, the remaining value was returned to the researcher. Participants were requested to eat the snack before continuing the behavioral tests. Food choices were photographed before and after the consumption and all the participants ate the snack. The receipt which included the selected products and the amount spent was saved and attached to the participant’s protocol. Subsequently, the amount spent, non-industrialized snacks recipes, and food choices were entered into an Excel^®^ data spreadsheet (Microsoft). For each participant, the nutritional composition of the selected snack was calculated with the aid of a table of household measures (24) and the USDA National Nutrient Database (25).

#### 2.4.2 MAVAN

Children and mothers were offered a test meal in the laboratory at approximately 10:30 a.m. including different types of foods in pre-weighed portions for 30 minutes. Pre-weighed plates of the different foods, chosen with the assistance of a nutritionist to represent local habitual snack items and to have similar colors, were displayed in a buffet to which the child had total access (26, 27). A table with two sets of plates was placed in the center of the room, with chairs for mother and child on both sides (facing each other) (**Supplementary Figure S2**). A cushion was placed on the child’s chair to facilitate accessibility of the different foods. Mothers were instructed to offer a light breakfast to participants at home beforehand and not to share plates or influence children’s choices. At the end of the session, the remaining foods were weighed again to measure the intake. Based on the nutritional content of each food and the amount eaten, we calculated the amount of fat, carbohydrates, and protein ingested (28).

### 2.5 Resting-State fMRI Acquisition and Data Processing

In both sites, the scan protocol included anatomical and resting-state fMRI acquisition. In the Brazilian sample, participants were scanned with at least 4 hours of fasting. About 30 minutes prior to the scan, they received a standardized snack [a cereal bar + one box juice = 174 kcal, carbohydrates 39g (90% of total calories), protein 0.9 g (2% of total calories) and 1.6 g of lipids (8% of total calories)]. Structural and functional images were collected on a 3.0T MRI scanner (GE Healthcare Signa HDxT) with an eight-channel head coil used for signal reception. Structural T1 weighted images were acquired for the whole cerebrum (1 mm^3^ isotropic voxels, 170 contiguous slices, 256 x 256 mm grid, TR=6.1ms, TE=2.18ms) during the first five minutes of the imaging session. Resting-state functional MRI was carried out by acquiring T2* Echo Planar Images (EPI) on a 7-minute-long run with the following parameters: TR=2000ms, TE=30ms, flip angle=90 degrees with 240mm x 240mm FOV with 26 interleaved axial slices of 4 mm on a 80x64 matrix.

In the Canadian sample, data were acquired using a 3T Trio Siemens scanner. High resolution T1-weighted images for the whole cerebrum (1 mm isotropic 3D MPRAGE, sagittal acquisition, 256 x 256 mm grid, TR=2300ms, TE=4ms, FA=9degrees) were obtained in an approximately five-minute scan. Resting-state fMRI was obtained on a 6-minute-long run of blood oxygenation level-dependent (BOLD) signal at rest using a gradient echo-planar imaging sequence with the following parameters: TR=2000 ms, TE=30 ms, flip angle=90 degrees with 3x3 mm in-plane resolution with 33 slices of 4 mm on a 64x64 matrix. During the resting-state functional MRI acquisition participants in both cohorts were instructed to stay with their eyes open and fixating on a “+” at the center of a screen. Participants were instructed to relax and avoid thinking about anything in particular.

Images were pre-processed using Statistical Parametric Mapping (SPM, v.8 for PROTAIA and v.12 for MAVAN, University College London, UK; http://www.fil.ion.ucl.ac.uk/spm in conjunction with the default processing pipeline using the CONN Functional Connectivity Toolbox (CONN toolbox; www.nitrc.org/projects/conn, RRID : SCR_009550).

First, the images were converted from DICOM (scanner format) to Nifti-1 format for processing. In order to compensate for different temporal offsets between slices during acquisition, temporal data interpolation (slice-timing correction) was applied to the functional images (29). After slice-timing correction, the images were processed to correct movement-related artifacts, the preprocessing pipeline developed by CONN (https://web.conn-toolbox.org/fmri-methods/preprocessing-pipeline) involves an outlier identification process *via* calculation of a framewise displacement each timepoint with a 140x180x115mm bounding box. If the framewise displacement exceeds 0.9mm, then this acquisition frame is flagged as a potential outlier. Furthermore, the global BOLD signal change is computed at each timepoint, and if the average BOLD signal changes greater than 5 s.d. then this is also flag the acquisition as an outlier. Each image was transformed *via* a 6-parameter rigid-body transformation, and then an autoregressive-moving average model was applied to correct for changes in head positions (30). Functional slice-time, motion corrected images were then co-registered to the individual raw T1 anatomical images (PROTAIA: Ashburner & Friston, 1997; MAVAN: Montreal Neurological Institute; MNI152). High-resolution anatomical images were segmented into grey matter, white matter and cerebrospinal fluid (CSF) (31); the smoothed gray matter images were then transformed into a standard space using a 12-parameter affine transformation and a 6-parameter three-dimensional quadratic deformation (32, 33). The parameters for the normalization to the standard space were applied to both anatomical and functional images for each subject. Following normalization, the functional data were smoothed using 8x8x8 mm Full Width at Half Maximum (FWHM) Gaussian kernel for statistical analysis (34). In PROTAIA, three subjects were excluded due to excess of movement during the resting-state fMRI, while in MAVAN five subjects were excluded due to excess movement and two for missing T1 data resulting in 40 and 49 total participants respectively.

Resting-state fMRI analysis was processed using CONN functional connectivity toolbox (35). Functional connectivity analysis was performed using a seed-driven approach with orbitofrontal cortex (OFC, right and left – Brodmann area 11) as seed points and adjusted for multiple comparisons. Functional imaging signal was filtered using a temporal band-pass filter of 0.01 Hz to 0.08 Hz. Residual motion correction parameters were used as regressors in the model according to the method described by Behzadi et al. (36) to mitigate motion-related artifacts in the seed-based connectivity analysis. The aCompCor method, part of the CONN pipeline, identifies confounding effects with noise from cerebral white matter and cerebrospinal components as well as head motion. Factors that are identified as confounds were then removed from volumes using Ordinary Least Square regression. Regions of interest (ROIs) corresponding to the ventral striatum, dorsal striatum, amygdala, medial prefrontal cortex, and dorsolateral prefrontal cortex were used to determine whether they were positively or negatively correlated with the OFC seed points (37). ROIs for seed points and target regions were extracted from Desikan et al. (38) and from the Harvard-Oxford subcortical structural atlases and comprehended only grey matter tissue (**Supplementary Figure S3**). For description of the OFC mask anatomic definition, including lateral and medial OFC, please see Desikan et al. (38). Individual Fisher’s Z scores from the OFC to the anticorrelated or positive correlated ROIs were calculated according to Weissenbacher et al. (39).

Seed points and ROIs were chosen based on the “appetitive network” (8) and on literature search. The OFC is a brain area related to motivation and compulsive behavior (40), its hyperactivity has been observed in individuals with obsessive compulsive disorder (41, 42). Bracht et al. (43) found that an increased OFC-NAcc functional connectivity is associated with craving in alcohol use disorder, and Black et al. (44) demonstrated greater resting-state functional connectivity between left middle frontal gyrus and left lateral OFC in obese children when compared to healthy weight children. Moreover, activity in the OFC and in the DL-PFC encodes subjects’ willingness to pay for food items (9). Therefore, it was assumed that SGA individuals would present connectivity changes between reward and self-control brain regions.

### 2.6 Statistical Analysis

Data were entered and analyzed in the Statistical Package for the Social Sciences (SPSS) 20.0 software (SPSS Inc., Chicago, IL, USA). Quantitative variables were described as mean ± standard error of the mean (SEM), whereas categorical data were described using absolute (n) and relative (%) frequencies. Comparisons between two groups were performed using Student’s t-test for parametric continuous variables or Kruskal-Wallis for non-parametric continuous variables and Chi-square test for categorical variables. The food choice-related data were analyzed by one-way ANOVA using BMI Z-Score and sex as covariates. The resting-state fMRI data were analyzed using the Student’s t-test in order to compare the connectivity between SGA individuals (BWR <0.85) and controls. Exploratory significance levels for all measures were set at p<0.05. To ensure reliability and robustness of exploratory significant mean differences we undertook further analyses to sample composition using 2,000 non-parametric bootstrapping with replacement and random resampling, in which 95% confidence intervals (Bootstrap CI) are reported. We also calculated false discovery rate (FDR) corrected q-values for each set of p-values from each resting-state fMRI seed points (45).

## 3 Results

### 3.1 Sample Description

**Figure 1:**
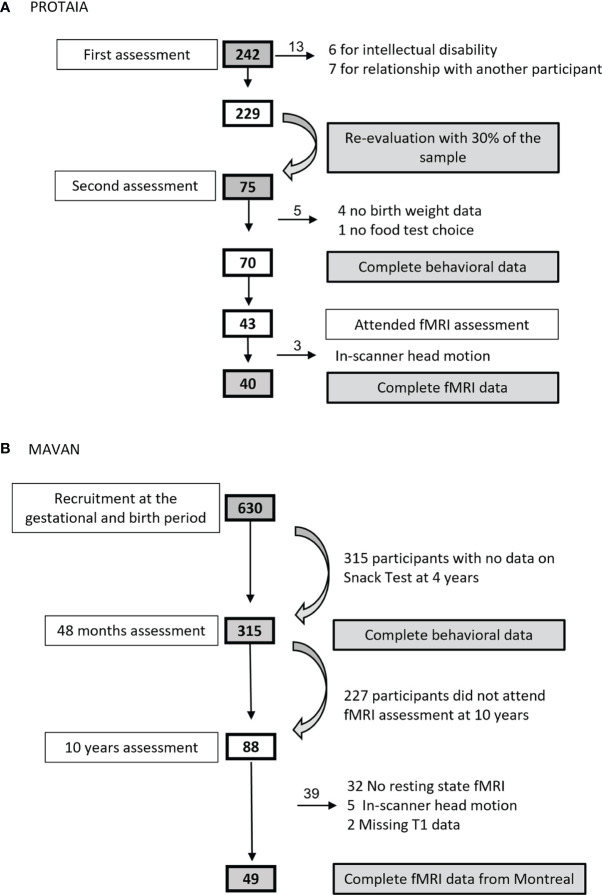
Sample size flowcharts from **(A)** PROTAIA and **(B)** MAVAN.

In the PROTAIA cohort, 75 healthy youths attended the behavioral data collection, however four could not inform birth data and one did not participate in the snack test, totaling 70 adolescents (SGA = 21.4%). Forty-three attended the fMRI visit, but data from 40 subjects (SGA = 17.5%) were processed and analyzed because three subjects had head excess movement. In the MAVAN cohort, 315 participated in the behavioral tests (SGA = 14.9%) and 49 were scanned for the fMRI analysis (SGA = 28.5%). **Figure 1** describes the sample sizes from both cohorts.

**Table 1** shows the participants’ characteristics from both cohorts. SGA and controls were not different regarding sex, color, maternal education, socioeconomic status, BMI, gestational age and prevalence of anxiety. As expected, the SGA group had a mean birth weight lower than the control group in both cohorts.

**Table 1:** Participants’ characteristics, according to the birth weight for gestational age groups (SGA vs. controls).

Behavioral tests samples				
	PROTAIA		MAVAN	
	Controls^$^ (n=55)	SGA^$^ (n=15)	Controls (n=268)	SGA (n=47)
Male^a^	21 (38.2%)	6 (40.0%)	142 (52.98%)	23 (48.93%)
White color^a^	36 (66.7%)	11 (73.3%)	189 (76.20%)	35 (77.77%)
Anxious^a^	24 (43.6%)	9 (60.0%)	———–	———–
Age (years)^b^	17.62 ± 0.32	17.08 ± 0.60	4.06 ± 0.01	4.05 ± 0.01
Birth weight (g)^b^	3286.55 ± 59.80	2457.33 ± 89.63*	3495.83 ± 24.18	2735.95 ± 36.38**
BMI^b^	23.62 ± 0.62	21.71 ± 1.10	16.09 ± 0.09	16.09 ± 0.32
BMI Z score^b^	0.60 ± 0.16	0.03 ± 0.32	0.53 ± 0.06	0.48 ± 0.19
Gestational age^c^	40.0 (38.0-40.0)	40.0 (37.0-40.0)	39.0 (38.0-40.0)	39.0 (39.0-40.0)
SES^d^	16.71 ± 0.66	18.39 ± 1.57	65 (24.8%)	11 (24.4%)
Maternal education^a^^,^ ^e^	11 (30.6%)	3 (33.3%)	55 (20.52%)	9 (19.56%)
**Resting-state fMRI connectivity samples**				
	**Controls^&^** **(n=33)**	**SGA^&^** **(n=7)**	**Controls** **(n=35)**	**SGA** **(n=14)**
Male^a^	15 (45.5%)	4 (57.1%)	17 (48.6%)	6 (42.9%)
White color^a^	20 (62.5%)	5 (71.4%)	27 (79.41%)	11 (78.57%)
Anxious^a^	11 (33.3%)	4 (57.1%)	————–	————
Age (years)^b^	17.72 ± 0.41	17.91 ± 0.99	10.1 ± 0.18	10.5 ± 0.17
Birth weight (g)^b^	3344.39 ± 62.15	2631.43 ± 63.75*	3363 ± 57.6	2624 ± 69.0**
BMI^b^	23.12 ± 0.64	21.67 ± 1.85	17.23 ± 0.55	17.89 ± 1.05
BMI Z score^b^	0.50 ± 0.17	-0.10 ± 0.56	-0.01 ± 0.16	0.19 ± 0.31
Gestational age^c^	40.0 (37.0-40.0)	40.0 (40.0-40.0)	39.0 (38.0-40.0)	39.0 (39.0-40.0)
SES^d^	17.75 ± 0.91	16.00 ± 1.98	8 (25%)	2 (16.6%)
Maternal education^a,e^	10 (45.5%)	0 (0.0%)	9 (25.7%)	4 (28.6%)

a Chi-square. Data expressed as absolute (n) and relative (%) frequencies.

b Student's t-test. Data expressed as mean ± SEM.

c Kruskal-Wallis test. Data expressed as median and interquartile range.

d Mean ± SEM ABEP score in PROTAIA and absolute (n) and relative (%) frequencies for household total gross income ≤ 40k a year in MAVAN.

e PROTAIA: ≤ 8 years of schooling; MAVAN: high school diploma or less.

*p<0.05; **p<0.001.

**^$^** Color: 54 controls, 15 SGA; Maternal education: 36 controls, 9 SGA.

**^&^** Color: 32 controls, 7 SGA; Maternal education: 22 controls, 4 SGA.

### 3.2 Feeding Behavior Assessment

Snack test data are shown in **Table 2**. In the PROTAIA cohort, a difference between groups was observed in the amount of the financial resource used to buy the snack. The SGA group used a smaller quantity, although the total caloric intake was comparable between groups [F(1,65)=1.91; p=0.172]. In both cohorts, the macronutrients’ intake in grams and percentage of total calories were not different between SGA and controls. The same comparisons were performed dividing the energy and nutrients’ consumption per body weight in kg and no differences between groups were observed in both cohorts (**Supplementary Table S1**).

**Table 2:** Food choice tests’ data, according to birth weight for gestational age group.

	PROTAIA		MAVAN	
Variables	Controls (n=55)	SGA (n=15)	Controls (n=268)	SGA (n=47)
Amount spent (R$)	8.54 ± 0.27	7.22 ± 0.52 *	————	————
Energy (kcal)	554.89 ± 27.16	480.01 ± 52.61	334.86 ± 9.1	301.55 ± 12.35
CHO (g)	69.91 ± 3.75	63.37 ± 7.27	40.40 ± 1.26	37.33 ± 2.09
CHO (% total kcal)	51.12 ± 1.50	52.65 ± 2.91	49.00 ± 1.00	50.00 ± 2.00
Sugar (g)	31.37 ± 2.95	30.16 ± 5.71	22.31 ± 0.72	20.4 ± 1.15
Fiber (g)	2.74 ± 0.24	2.37 ± 0.46	1.93 ± 0.11	1.93 ± 0.18
PTN (g)	17.76 ± 1.31	12.65 ± 2.53	12.21 ± 0.38	11.09 ± 0.54
PTN (% total kcal)	12.35 ± 0.67	10.57 ± 1.30	15.00 ± 0.00	15.00 ± 1.00
FAT (g)	23.15 ± 1.31	19.91 ± 2.54	13.62 ± 0.49	11.86 ± 0.63
FAT (% total kcal)	37.18 ± 1.17	37.24 ± 2.26	36.00 ± 1.00	35.00 ± 1.00

One-way ANOVA with BMI Z-Score and sex as covariates; data expressed as mean ± SEM.

*p<0.05. CHO, carbohydrate; PTN, protein.

Exploratory analyses of the snack test according to sociodemographic data, as well as the correlations between snack test results (original data and divided per body weight) and resting-state fMRI connectivity are presented in the Supplemental data (**Tables S2A–D**, **S3A–F** and **S4A–F**).

### 3.3 Resting-State fMRI Connectivity

**Figure 2:**
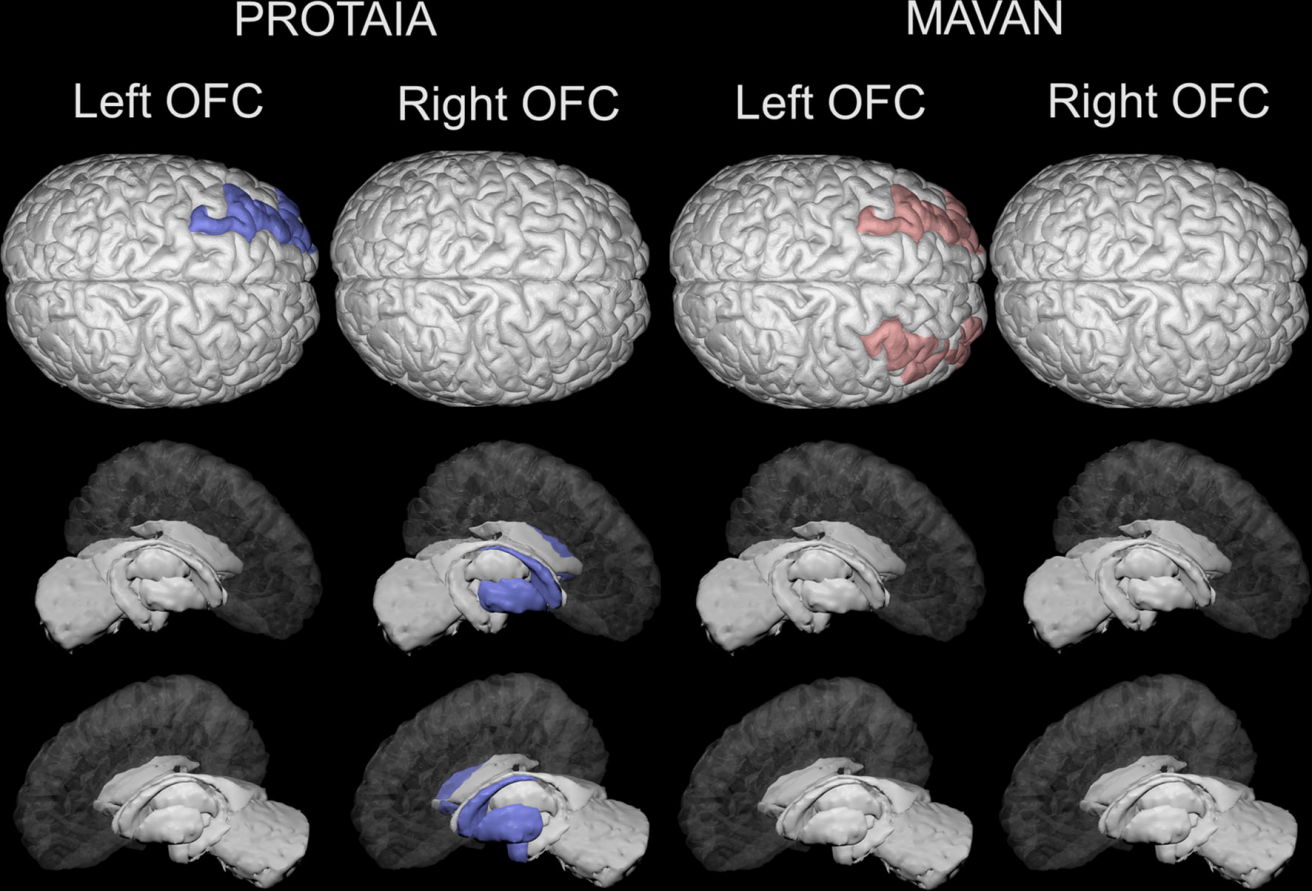
Brain areas depicting statistically significant differences in resting-state functional connectivity (rsFC) with the left and right orbital frontal cortex between SGA and controls in the PROTAIA and MAVAN cohorts. Blue color (left DL-PFC, left amygdala and right and left dorsal striatum) represents lower rsFC in SGA versus Controls. Red color (right and left DL-PFC) represents higher rsFC in SGA versus Controls.

The brain imaging results are shown in **Table 3** and **Figure 2**.

**Table 3:** SGA versus controls rs-FC between orbitofrontal cortex (left and right) and ROIs with significant results in one of the cohorts.

	PROTAIA		MAVAN	
	left OFC	right OFC	left OFC	right OFC
left DS	SGA: 0.028 ± 0.055	SGA: -0.098 ± 0.060	SGA: 0.25 ± 0.06	SGA: 0.18 ± 0.06
	Controls: 0.059 ± 0.034	Controls: 0.061± 0.030	Controls: 0.20 ± 0.03	Controls: 0.19 ± 0.03
	p=0.689	p=0.024*	p= 0.345	p=0.839
right DS	SGA: -0.051 ± 0.066	SGA: -0.101 ± 0.050	SGA: 0.07 ± 0.08	SGA: 0.22 ± 0.05
	Controls: 0.004 ± 0.036	Controls: 0.103 ± 0.030	Controls: 0.04 ± 0.04	Controls: 0.23 ± 0.03
	p=0.521	p=0.003*	p=0.725	p=0.939
left AMY	SGA: -0.066 ± 0.051	SGA: -0.129 ± 0.050	SGA: 0.30 ± 0.06	SGA: 0.10 ± 0.04
	Controls: 0.033 ± 0.040	Controls: 0.101 ± 0.040	Controls: 0.20 ± 0.04	Controls: 0.13 ± 0.03
	p=0.283	p= 0.008*	p=0.128	p=0.682
right AMY	SGA: -0.006 ± 0.072	SGA: 0.038 ± 0.042	SGA: 0.28 ± 0.07	SGA: 0.22 ± 0.07
	Controls: -0.027 ± 0.033	Controls: 0.095 ± 0.040	Controls: 0.14 ± 0.04	Controls: 0.18 ± 0.04
	p=0.790	p=0.528	p=0.061	p=0.642
left DL-PFC	SGA: 0.004 ± 0.060	SGA: -0.162 ± 0.091	SGA: 0.21 ± 0.06	SGA:0.19 ± 0.07
	Controls: 0.229 ± 0.040	Controls: -0.007 ± 0.041	Controls: 0.06 ± 0.04	Controls: 0.08 ± 0.04
	p=0.019*	p=0.119	p=0.036*	p=0.161
right DL-PFC	SGA: -0.056 ± 0.052	SGA: -0.031 ± 0.060	SGA:0.01 ± 0.09	SGA:0.17 ± 0.08
	Controls: 0.092 ± 0.045	Controls: 0.011 ± 0.052	Controls: -0.14 ± 0.03	Controls:0.12 ± 0.04
	p=0.147	p=0.719	p=0.045*	p=0.531

Student’s t-test. Data expressed as mean ± SEM (*p<0.05). OFC, orbitofrontal cortex; DL-PFC, dorsolateral prefrontal cortex; AMY, amygdala; DS, dorsal striatum.

In the PROTAIA cohort, the SGA group exhibited predominately negative resting-state functional connectivity (rs-FC) between a network of areas that included the right OFC and the left (mean difference = 0.159, Bootstrap CI = 0.05: 0.30, FDR q = 0.048) and right dorsal striatum (mean difference = 0.204, Bootstrap CI = 0.09: 0.32, FDR q = 0.018), and the left amygdala (mean difference = 0.230, Bootstrap CI = 0.12: 0.35, FDR q = 0.024), while in controls the rs-FC between these areas was predominately positive. Also, the SGA group compared to controls showed lower connectivity between the left OFC and the left DL-PFC (mean difference = 0.225, Bootstrap CI = 0.10: 0.36, FDR q = 0.024).

In the MAVAN cohort, the differences in functional connectivity were found between left OFC and both sides of the DL-PFC. The SGA group had higher levels of connectivity between left OFC and left DL-PFC in comparison to controls. This difference was significant according to the bootstrap procedure (mean difference =0.15, Bootstrap CI = 0.01: 0.30), but it was not significant when adjusting for multiple testing (FDR q = 0.12). Furthermore, the SGA group also showed higher connectivity between the left OFC and the right DL-PFC in relation to the control group but this result did not met the Bootstrap and FDR thresholds (mean difference =0.15, Bootstrap CI = -0.03: 0.30, FDR q = 0.12).

## 4 Discussion

The current study showed that SGA individuals have altered connectivity between the OFC and the DL-PFC in the two samples, and between the OFC, dorsal striatum and amygdala in the adolescent sample. The direction of the association between SGA and the OFC-DL-PFC connectivity was different in children and adolescents. Although there were no differences between SGA and controls regarding anthropometric measures and snack nutrients’ consumption in both cohorts, SGA adolescents exhibited a different behavior when buying a snack, spending less money without eating less calories. The results confirm the hypothesis that SGA is associated with changes in food-related behaviors, and introduces the idea that SGA individuals, when compared to controls, have a different brain resting-state connectivity in areas related to reward and decision-making.

In both samples, the food choice test showed no differences in food preference between SGA and controls, since the percentage of calories from carbohydrates, proteins and lipids did not differ between groups. In the current study, the food preference was assessed by the spontaneous choice of food in a single meal in a controlled environment (laboratory or clinical center cafeteria), which may not have been able to detect broader differences in food preference as described in the literature in different ages (46–51).

Confirming our hypothesis, altered rs-FC between the OFC and brain regions involved in encoding the subjective value for available goods and decision-making was observed in SGA groups when compared to controls. SGA adolescents exhibited decreased rs-FC between the OFC and the DL-PFC, a circuit encoding subjects’ willingness to pay for food items (9). Interestingly, at the behavioral level, SGA adolescents spent less money on food than controls (even being informed that they would need to return the non-used money), but no significant differences between the quantity of calories consumed was found. This suggests that they choose cheaper (and usually nutritionally poorer) foods, as previously proposed (52, 53). The functional connectivity between the DL-PFC and the other frontal-lobe regions is associated with attribution of value at the time of a behavioral choice (54). It has been suggested that these areas are involved in foreseeing potential outcomes from not chosen actions (55), an ability that may be impaired in SGA individuals. Surprisingly, the same pattern of decreased connectivity between the OFC, DL-PFC and amygdala, observed in SGA adolescents, is seen in schizophrenia patients (56). Lower connectivity in schizophrenic patients is associated with executive function social cognition deficits – a deficit that has also been described in the SGA population (57).

However, the altered associations between OFC - DL-PFC rs-FC in SGA were in the opposite way in children and adolescents. SGA children exhibited higher rs-FC between the OFC and the DL-PFC than controls, while SGA adolescents showed lower rs-FC. The differences could be related to the demographic dissimilarities in the two populations (see **Table 1**). In addition, studies with preterm children and children exposed to early life stress have also identified positive correlations between frontal and dorsolateral regions. For example, in an independent component analysis higher correlations of the left motor cortex and the bilateral posterior temporal cortex were observed in pre-term infants at 18 and 36 months of age, while early stress was positively associated with temporal similarity of a cluster of voxels in the left middle frontal gyrus (56, 57). Alterations of connectivity strength and direction from childhood to adolescence could be reflecting the maturation of cognitive networks. For example, the connectivity of important networks like the lateral frontoparietal network and the default mode network can be positively correlated in childhood, anti-correlated in adolescence, and negatively correlated in young adults (58). The differences observed for SGA children could be altering the fine-tuning of networks that are dynamic, highly dependent on the interaction between the genetic substrate and quality of the prenatal and early environment resulting in weaker connectivity in adolescence (59–61). Longitudinal studies are therefore necessary to test this hypothesis.

In addition, other altered rs-FC networks were observed in SGA adolescents, who exhibited food-related altered behavior. The rs-FC between right OFC and left amygdala was predominately negative in SGA, while predominately positive in controls. It is interesting to note that it is described in the literature a negative association between the OFC-amygdala functional connectivity and impulsivity scores in healthy subjects (62). As impulsivity seems to be an important behavioral feature linking impaired fetal growth to altered feeding behavior and preferences over the life-course (63, 64), the diminished connectivity between these two areas described in the current study could indeed characterize this group and be involved in the way they behave towards food. Finally, considering the proposed view of overeating palatable foods as a “food addiction” (65), the altered brain connectivity between the OFC, striatum and amygdala seen in our SGA adolescent group resembles the pattern previously seen in alcohol abusers (66, 67), cocaine users (68) and Internet Gaming Disorder (IGD) youths (69, 70).

Although our study has identified neurobehavioral changes in individuals who were born SGA, they had normal BMI both in childhood and adolescence. This suggests that the neurobehavioral changes precede the development of the well described increased adiposity and its metabolic consequences in these individuals (71, 72). More evident anthropometric and metabolic disturbances may arise over the years in this population with the cumulative exposure to particular food cues, altered behavior towards food (e.g. when purchasing foods), and consequent intake of energy-dense foods with low nutritional quality.

Our study was able to look for similarities in feeding behavior and rs-FC in two cohorts of SGA individuals, bringing results from different life stages. Our objective of having the two samples in the study was not to have a comparison between them, but rather pursuing a replication of the findings in an independent cohort. We understand that replication may be challenging, and perfect harmonization of the samples age, predictors and outcomes is virtually impossible. Even with these limitations, the effort for replication of the findings within the same study is aligned with the best methodological practices and we believe this is a strength of our study, considering the replication crisis in research (73, 74). Also, at first glance, the SGA groups seem to have a small sample size, however our study reflects the percentage of SGA in the population (10-20%). Despite this, we were still able to find brain rs-FC differences between groups in both cohorts. We suggest that the specific pattern of brain rs-FC observed could be leaving SGA individuals in a vulnerable position when facing food options on a daily basis. The increased vulnerability may affect food-related behaviors, as seen here, and food preferences reported in many other studies (46–51, 75–79), and play a role in the long-term risk for developing chronic non-communicable diseases. Therefore, the current study adds to the literature demonstrating that while SGA individuals are more prone to future impairments in both physical and mental health in the adult years, they may represent an important populational group for targeted, well-matched intervention.

## Data Availability Statement

The datasets presented in this article are not readily available because they contain sensitive information. Requests to access the datasets should be directed to Patricia P. Silveira.

## Ethics Statement

The studies involving human participants were reviewed and approved by PROTAIA: Comitê de Ética em Pesquisa do Hospital de Clínicas de Porto Alegre (HCPA). MAVAN: Institutional Review Boards at hospitals and university affiliates: McGill University, l’Universite de Montreal, the Royal Victoria Hospital, Jewish General Hospital, Centre Hopitalier de l’Universite de Montreal, Hôpital Maisonneuve-Rosemont, St Joseph’s Hospital, and McMaster University. Written informed consent to participate in this study was provided by the participants’ legal guardian/next of kin.

## Author Contributions

RDM, GS, MM, RL, GM, and PS were responsible for the study concept and design. RDM, TM, RR, DR, AM, RT, and ABo contributed to the acquisition of clinical data. RMD, EM, LM, AF, ABu, and SB assisted with fMRI data acquisition and/or analysis. RMD, EM, and PS contributed to the statistical analysis of the study. RDM and EM drafted the manuscript and PS and MM provided critical revisions on the manuscript for important intellectual content. All authors contributed to the article and approved the submitted version.

## Funding

Universal National Counsel of Technological and Scientific Development (CNPq) (Silveira PP, 478820/2010-0); Foundation for the Coordination of Higher Education and Graduate Training (PNPD/CAPES, 3024/2010); Fundo de Incentivo à Pesquisa (FIPE/HCPA), Canadian Institutes of Health Research (CIHR) [PJT-166066 and PJT - 173237 to PI PPS], and the JPB Foundation through a grant to the JPB Research Network on Toxic Stress: A Project of the Center on the Developing Child at Harvard University. Dr. Levitan acknowledges support from the Cameron Holcombe Wilson Chair in Depression studies, CAMH and University of Toronto.

## Conflict of Interest

The authors declare that the research was conducted in the absence of any commercial or financial relationships that could be construed as a potential conflict of interest.

## Publisher’s Note

All claims expressed in this article are solely those of the authors and do not necessarily represent those of their affiliated organizations, or those of the publisher, the editors and the reviewers. Any product that may be evaluated in this article, or claim that may be made by its manufacturer, is not guaranteed or endorsed by the publisher.
