# Supplementary material for: Thrifty-Eating Behavior Phenotype at the Food Court – Programming Goes Beyond Food Preferences
Source: Front Endocrinol (Lausanne). 2022 May 23;13:882532. doi: 10.3389/fendo.2022.882532 (PMC9168906; doi:10.3389/fendo.2022.882532)
Supplement: Supplementary file 1 [file DataSheet_1.docx]

Supplementary Material

| 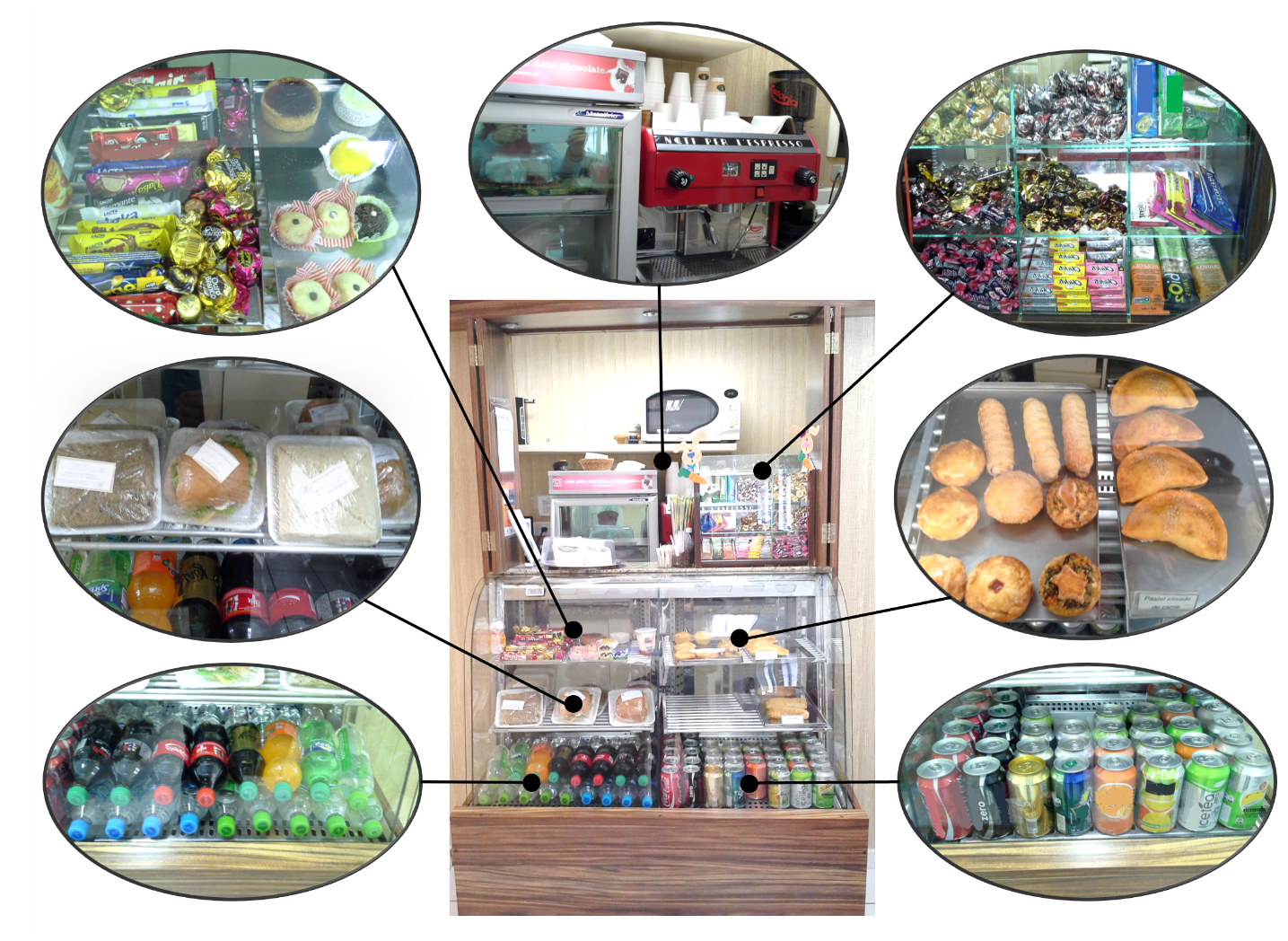 |
| --- |
| **Supplementary Figure S1.** **PROTAIA snack test.** Cafeteria of the Clinical Research Center with its food options. All the participants received a voucher with the same monetary value to purchase snacks of their choice. If the participant did not use the full value of the voucher, the remaining value was returned to the researcher. |

| 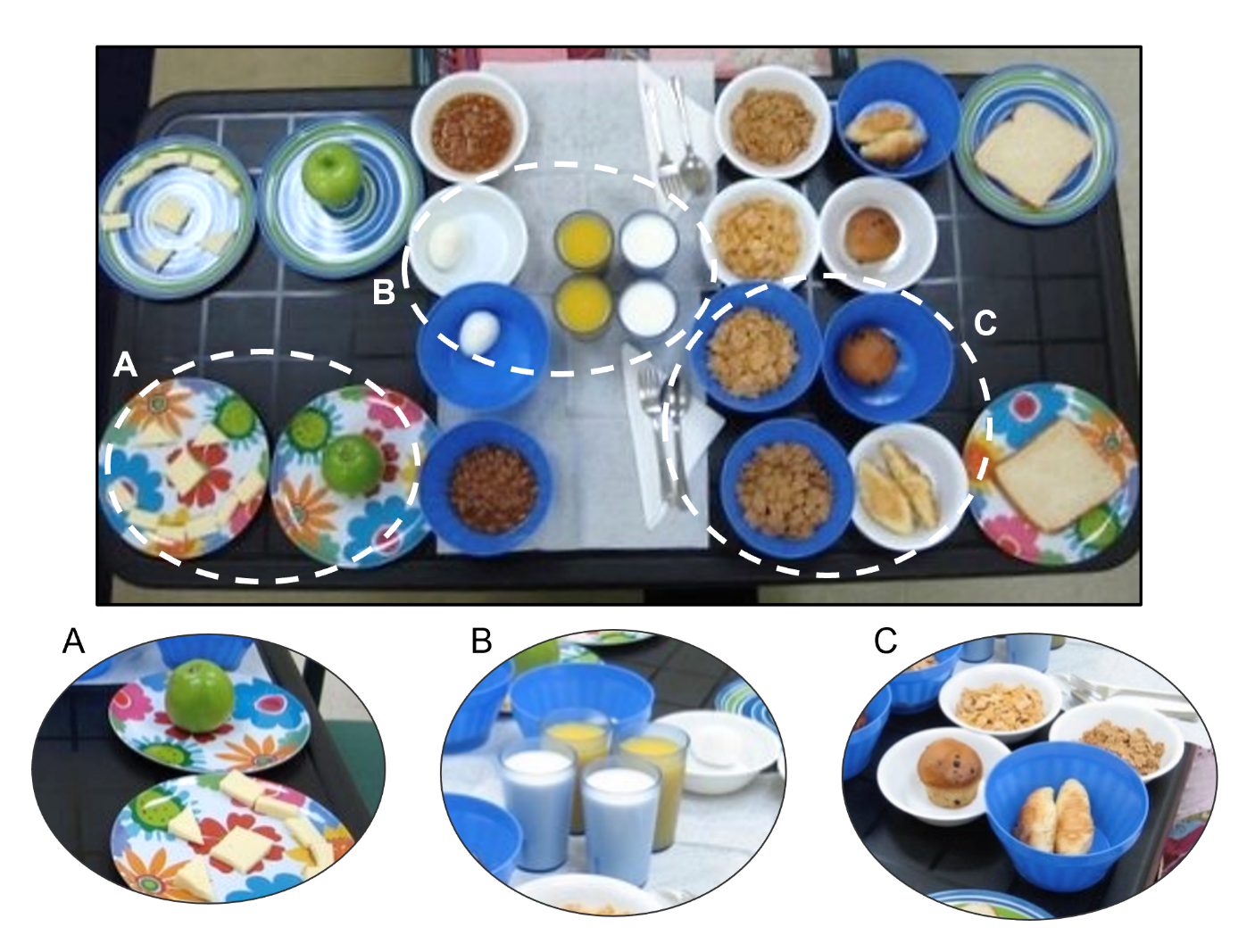 |
| --- |
| **Supplementary Figure S2.** **MAVAN snack test.** Laboratory test meal seen from above and some served foods in detail inside the circles. A table with two sets of plates was placed in the center of the room, with chairs for mother and child on both sides (facing each other). The portions served were pre-weighed, and at the end of the session, the remaining foods were weighed to measure the intake. |

**
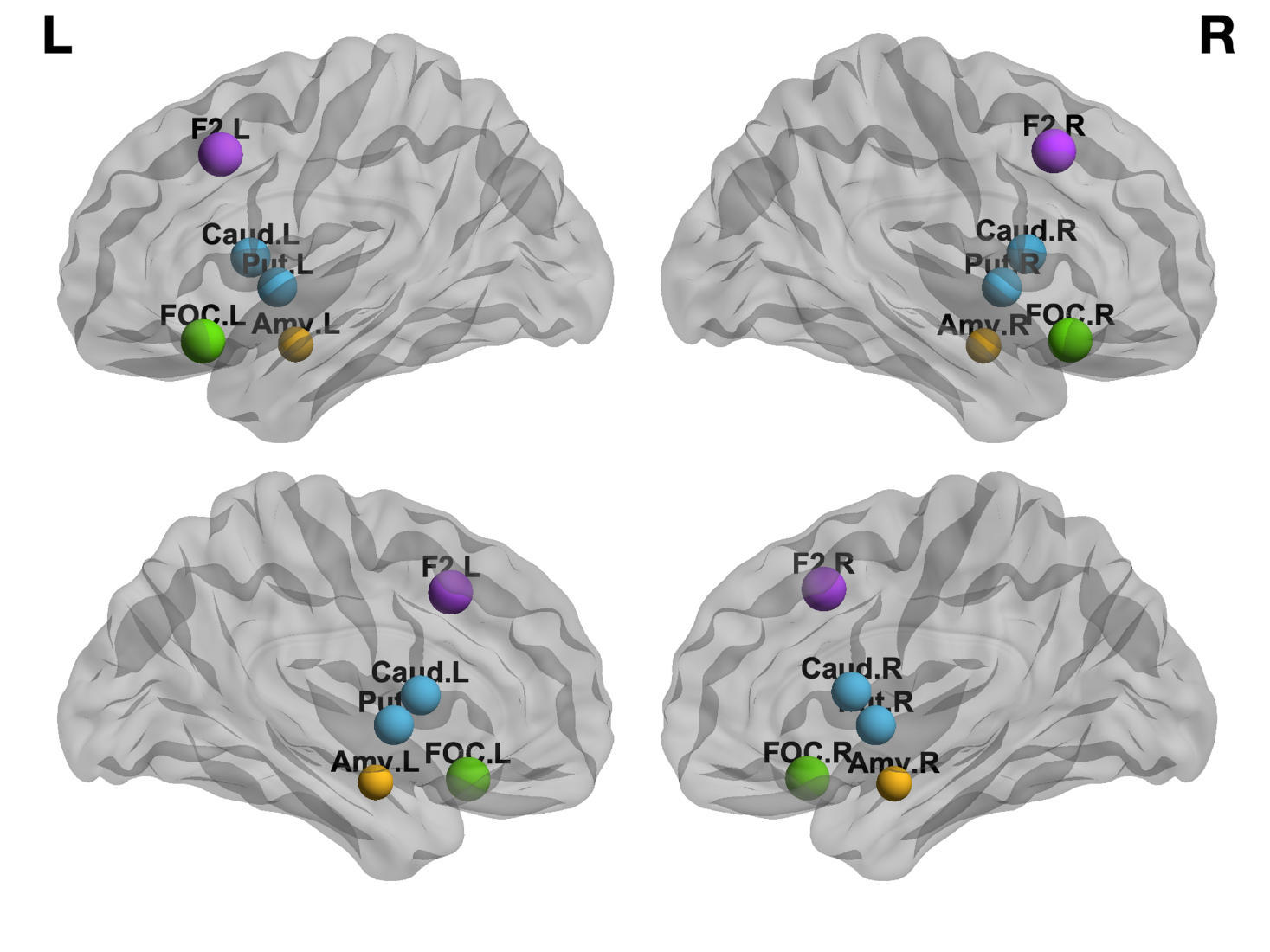
**

**Supplementary Figure S3.** **Illustrative brain images showing the seed (orbitofrontal cortex) in green and ROIs: amygdala in orange, dorsal striatum in blue and dorsolateral prefrontal cortex in purple**. The brain areas were created using the Harvard-Oxford atlas and visualized with the BrainNet Viewer (Xia et al., 2013, <http://www.nitrc.org/projects/bnv/>).

Xia M, Wang J, He Y (2013) BrainNet Viewer: A Network Visualization Tool for Human Brain Connectomics. PLoS ONE 8: e68910.

**Supplementary Table S1** - Food choice tests’ results expressed according to body weight.

|  | **PROTAIA** | | **MAVAN** | |
| --- | --- | --- | --- | --- |
| **Variables** | **Controls**  **(n=55)** | **SGA**  **(n=15)** | **Controls**  **(n=268)** | **SGA**  **(n=47)** |
| Energy (kcal/kg) | 8.74±0.48 | 8.31±0.91 | 19.05 ±0.44 | 18.5 ±1.21 |
| CHO (g/kg) | 1.10±0.06 | 1.09±0.12 | 2.31 ±0.06 | 2.28 ±0.19 |
| Sugar (g/kg) | 0.50±0.05 | 0.53±0.09 | 1.27 ±0.04 | 1.25 ±0.11 |
| Fiber (g/kg) | 0.43±0.01 | 0.39±0.01 | 0.11 ±0.01 | 0.11 ±0.01 |
| PTN (g/kg) | 0.28±0.02 | 0.22±0.04 | 0.70 ±0.02 | 0.68 ±0.05 |
| FAT (g/kg) | 0.36±0.02 | 0.35±0.04 | 0.77 ±0.02 | 0.74 ±0.06 |

One-way ANOVA with sex as covariate; data expressed as mean ± SEM

*p<0.05. CHO: carbohydrate; PTN: protein.

**Supplementary Table S2 -** Food choice tests’ results according to maternal education and BMI-for-age Z-scores in PROTAIA (A and B) and MAVAN (C and D). Student’s t-test performed for the total sample, control group and SGA group.

| **(A)** PROTAIA | | **TOTAL SAMPLE** | | | | **CONTROL** | | | | **SGA** | | | |
| --- | --- | --- | --- | --- | --- | --- | --- | --- | --- | --- | --- | --- | --- |
| **Maternal**  **education** | | N | Mean | Std. Error Mean | p-value | N | Mean | Std. Error Mean | p-value | N | Mean | Std. Error Mean | p-value |
| Amount spent (R$) | lower | 14 | 8.15 | .664 | .913 | 11 | 9.15 | .448 | .274 | 3 | 4.46 | 1.026 | .130 |
|  | higher | 31 | 8.23 | .380 |  | 25 | 8.41 | .395 |  | 6 | 7.46 | 1.102 |  |
| Energy (kcal) | lower | 14 | 552.69 | 61.034 | .923 | 11 | 610.74 | 66.055 | .516 | 3 | 339.86 | 64.634 | .454 |
|  | higher | 31 | 545.83 | 38.310 |  | 25 | 562.32 | 39.484 |  | 6 | 477.13 | 114.659 |  |
| PTN (g) | lower | 14 | 14.63 | 2.546 | .337 | 11 | 16.86 | 2.853 | .750 | 3 | 6.46 | 2.208 | .178 |
|  | higher | 31 | 17.68 | 1.767 |  | 25 | 17.99 | 1.961 |  | 6 | 16.38 | 4.418 |  |
| PTN (% total kcal) | lower | 14 | 10.15 | 1.223 | .108 | 11 | 10.97 | 1.439 | .466 | 3 | 7.13 | 1.256 | **.013*** |
|  | higher | 31 | 12.62 | .848 |  | 25 | 12.26 | .973 |  | 6 | 14.12 | 1.679 |  |
| CHO (g) | lower | 14 | 71.05 | 9.218 | .809 | 11 | 79.99 | 10.145 | .369 | 3 | 38.29 | 2.710 | .334 |
|  | higher | 31 | 68.76 | 4.781 |  | 25 | 70.89 | 4.941 |  | 6 | 59.89 | 14.173 |  |
| Sugar (g) | lower | 14 | 31.69 | 7.227 | .936 | 11 | 37.50 | 8.147 | .625 | 3 | 10.40 | 8.884 | .192 |
|  | higher | 31 | 32.28 | 3.689 |  | 25 | 33.37 | 4.272 |  | 6 | 27.77 | 7.194 |  |
| Fiber (g) | lower | 14 | 2.81 | .551 | .572 | 11 | 3.32 | .615 | .187 | 3 | .94 | .186 | .260 |
|  | higher | 31 | 2.50 | .271 |  | 25 | 2.53 | .285 |  | 6 | 2.39 | .803 |  |
| CHO (% total kcal) | lower | 14 | 51.29 | 3.048 | 1.000 | 11 | 51.90 | 2.840 | .970 | 3 | 49.03 | 11.436 | .976 |
|  | higher | 31 | 51.29 | 1.811 |  | 25 | 51.76 | 2.120 |  | 6 | 49.31 | 3.283 |  |
| FAT (g) | lower | 14 | 23.65 | 2.552 | .783 | 11 | 25.27 | 2.739 | .633 | 3 | 17.73 | 6.129 | .839 |
|  | higher | 31 | 22.71 | 1.960 |  | 25 | 23.49 | 2.133 |  | 6 | 19.50 | 5.058 |  |
| FAT (% total kcal) | lower | 14 | 38.95 | 2.573 | .426 | 11 | 37.70 | 2.091 | .723 | 3 | 43.52 | 10.367 | .438 |
|  | higher | 31 | 36.76 | 1.423 |  | 25 | 36.67 | 1.673 |  | 6 | 37.11 | 2.603 |  |

| **(B)** PROTAIA | | **TOTAL SAMPLE** | | | | **CONTROL** | | | | **SGA** | | | |
| --- | --- | --- | --- | --- | --- | --- | --- | --- | --- | --- | --- | --- | --- |
| **BMI Z-score** | | N | Mean | Std. Error Mean | p-value | N | Mean | Std. Error Mean | p-value | N | Mean | Std. Error Mean | p-value |
| Amount spent (R$) | normal | 45 | 8.31 | .304 | .734 | 32 | 8.80 | .273 | .190 | 13 | 7.11 | .729 | .639 |
|  | overweight | 25 | 8.14 | .409 |  | 23 | 8.15 | .439 |  | 2 | 8.05 | 1.150 |  |
| Energy (kcal) | normal | 45 | 532.72 | 30.005 | .734 | 32 | 554.51 | 33.642 | .925 | 13 | 479.09 | 62.396 | .821 |
|  | overweight | 25 | 549.86 | 40.180 |  | 23 | 559.51 | 41.434 |  | 2 | 438.86 | 191.270 |  |
| PTN (g) | normal | 45 | 16.54 | 1.472 | .894 | 32 | 18.35 | 1.792 | .603 | 13 | 12.10 | 2.187 | .569 |
|  | overweight | 25 | 16.87 | 1.898 |  | 23 | 16.94 | 1.941 |  | 2 | 16.00 | 11.285 |  |
| PTN (% total kcal) | normal | 45 | 11.99 | .772 | .951 | 32 | 12.65 | .947 | .570 | 13 | 10.37 | 1.251 | .570 |
|  | overweight | 25 | 11.91 | .895 |  | 23 | 11.87 | .925 |  | 2 | 12.47 | 4.845 |  |
| CHO (g) | normal | 45 | 68.03 | 4.091 | .847 | 32 | 70.21 | 4.463 | .988 | 13 | 62.68 | 9.077 | .944 |
|  | overweight | 25 | 69.36 | 5.579 |  | 23 | 70.09 | 5.925 |  | 2 | 60.93 | 19.395 |  |
| Sugar (g) | normal | 45 | 30.56 | 3.221 | .775 | 32 | 30.60 | 3.669 | .721 | 13 | 30.45 | 6.787 | .776 |
|  | overweight | 25 | 32.10 | 4.237 |  | 23 | 32.69 | 4.587 |  | 2 | 25.25 | 3.315 |  |
| Fiber (g) | normal | 45 | 2.57 | .257 | .569 | 32 | 2.66 | .273 | .596 | 13 | 2.33 | .600 | .749 |
|  | overweight | 25 | 2.82 | .372 |  | 23 | 2.91 | .392 |  | 2 | 1.80 | 1.245 |  |
| CHO (% total kcal) | normal | 45 | 51.71 | 1.769 | .783 | 32 | 51.73 | 2.074 | .613 | 13 | 51.67 | 3.517 | .454 |
|  | overweight | 25 | 50.95 | 1.826 |  | 23 | 50.25 | 1.848 |  | 2 | 59.05 | 8.065 |  |
| FAT (g) | normal | 45 | 21.97 | 1.472 | .579 | 32 | 22.63 | 1.734 | .584 | 13 | 20.33 | 2.838 | .485 |
|  | overweight | 25 | 23.31 | 1.859 |  | 23 | 24.06 | 1.879 |  | 2 | 14.71 | 7.930 |  |
| FAT (% total kcal) | normal | 45 | 36.84 | 1.326 | .649 | 32 | 36.20 | 1.481 | .274 | 13 | 38.41 | 2.845 | .213 |
|  | overweight | 25 | 37.81 | 1.551 |  | 23 | 38.62 | 1.556 |  | 2 | 28.49 | 3.850 |  |

| **(C)** MAVAN | | **TOTAL SAMPLE** | | | | **CONTROL** | | | | **SGA** | | | |
| --- | --- | --- | --- | --- | --- | --- | --- | --- | --- | --- | --- | --- | --- |
| **Maternal**  **Education** | | N | Mean | Std. Error Mean | p-value | N | Mean | Std. Error Mean | p-value | N | Mean | Std. Error Mean | p-value |
| Energy (kcal) | lower | 64 | 309.54 | 13.67 | 0.324 | 55 | 307.21 | 15.54 | 0.185 | 9 | 323.77 | 23.98 | 0.55 |
|  | higher | 250 | 327.53 | 8.49 |  | 213 | 333.26 | 9.11 |  | 37 | 294.52 | 22.84 |  |
| PTN (g) | lower | 64 | 11.56 | 0.65 | 0.640 | 55 | 11.39 | 0.72 | 0.377 | 9 | 12.61 | 1.52 | 0.28 |
|  | higher | 250 | 11.92 | 0.35 |  | 213 | 12.14 | 0.39 |  | 37 | 10.67 | 0.77 |  |
| PTN (% total kcal) | lower | 64 | 15.00 | 1.00 | 0.767 | 55 | 15.00 | 1.00 | 0.768 | 9 | 15.00 | 1.00 | 0.97 |
|  | higher | 250 | 15.00 | 1.00 |  | 213 | 15.00 | 0.0 |  | 37 | 15.00 | 1.00 |  |
| CHO (g) | lower | 64 | 36.21 | 1.91 | 0.145 | 55 | 36.48 | 2.2 | 0.144 | 9 | 34.6 | 2.56 | 0.76 |
|  | higher | 250 | 40.05 | 1.23 |  | 213 | 40.63 | 1.32 |  | 37 | 36.76 | 3.4 |  |
| Sugar (g) | lower | 64 | 20.56 | 1.19 | 0.370 | 55 | 20.61 | 1.33 | 0.314 | 9 | 20.26 | 2.64 | 0.97 |
|  | higher | 250 | 21.89 | 0.68 |  | 213 | 22.2 | 0.73 |  | 37 | 20.09 | 1.95 |  |
| Fiber (g) | lower | 64 | 1.68 | 0.14 | 0.213 | 55 | 1.75 | 0.16 | 0.321 | 9 | 1.24 | 0.2 | 0.26 |
|  | higher | 250 | 1.98 | 0.11 |  | 213 | 2.02 | 0.13 |  | 37 | 1.73 | 0.2 |  |
| CHO (% total kcal) | lower | 64 | 48.00 | 2.00 | 0.683 | 55 | 49.00 | 2.00 | 0.866 | 9 | 45.00 | 5.00 | 0.49 |
|  | higher | 250 | 49.00 | 1.00 |  | 213 | 49.00 | 1.00 |  | 37 | 49.00 | 2.00 |  |
| FAT (g) | lower | 64 | 12.99 | 0.77 | 0.893 | 55 | 12.68 | 0.84 | 0.513 | 9 | 14.87 | 1.96 | 0.18 |
|  | higher | 250 | 13.12 | 0.45 |  | 213 | 13.38 | 0.5 |  | 37 | 11.61 | 1.04 |  |
| FAT (% total kcal) | lower | 64 | 37.00 | 1.00 | 0.472 | 55 | 36.00 | 1.00 | 0.683 | 9 | 40.00 | 4.00 | 0.37 |
|  | higher | 250 | 36.00 | 1.00 |  | 213 | 36.00 | 1.00 |  | 37 | 36.00 | 2.00 |  |

| **(D)** MAVAN | | **TOTAL SAMPLE** | | | | **CONTROL** | | | | **SGA** | | | |
| --- | --- | --- | --- | --- | --- | --- | --- | --- | --- | --- | --- | --- | --- |
| **BMI Z-score** | | N | Mean | Std. Error Mean | p-value | N | Mean | Std. Error Mean | p-value | N | Mean | Std. Error Mean | p-value |
| Energy (kcal) | normal | 290 | 323.20 | 7.67 | 0.394 | 248 | 326.42 | 8.17 | 0.696 | 42 | 304.21 | 21.93 | 0.265 |
|  | overweight | 21 | 348.66 | 27.97 |  | 17 | 339.2 | 31.99 |  | 4 | 388.88 | 60.92 |  |
| PTN (g) | normal | 290 | 11.83 | 0.32 | 0.488 | 248 | 11.95 | 0.35 | 0.759 | 42 | 11.11 | 0.79 | 0.284 |
|  | overweight | 21 | 12.70 | 1.14 |  | 17 | 12.39 | 1.33 |  | 4 | 14.05 | 2.18 |  |
| PTN (% total kcal) | normal | 290 | 15.00 | 1.00 | 0.970 | 248 | 15.00 | 0.00 | 0.911 | 42 | 15.00 | 1.00 | 0.839 |
|  | overweight | 21 | 15.00 | 1.00 |  | 17 | 15.00 | 1.00 |  | 4 | 15.00 | 1.00 |  |
| CHO (g) | normal | 290 | 39.35 | 1.11 | 0.619 | 248 | 39.66 | 1.17 | 0.952 | 42 | 37.5 | 3.42 | 0.362 |
|  | overweight | 21 | 41.49 | 3.74 |  | 17 | 39.94 | 4.41 |  | 4 | 48.07 | 6.16 |  |
| Sugar (g) | normal | 290 | 21.56 | 0.63 | 0.203 | 248 | 21.69 | 0.65 | 0.314 | 42 | 20.78 | 2.02 | 0.436 |
|  | overweight | 21 | 24.67 | 2.24 |  | 17 | 24.33 | 2.7 |  | 4 | 26.1 | 3.36 |  |
| Fiber (g) | normal | 290 | 1.96 | 0.1 | 0.316 | 248 | 2.01 | 0.12 | 0.109 | 42 | 1.67 | 0.21 | 0.150 |
|  | overweight | 21 | 1.57 | 0.2 |  | 17 | 1.29 | 0.16 |  | 4 | 2.74 | 0.63 |  |
| CHO (% total kcal) | normal | 290 | 49.00 | 1.00 | 0.807 | 248 | 49.00 | 1.00 | 0.696 | 42 | 49.00 | 2.00 | 0.789 |
|  | overweight | 21 | 48.00 | 2.00 |  | 17 | 48.00 | 3.00 |  | 4 | 51.00 | 3.00 |  |
| FAT (g) | normal | 290 | 13.01 | 0.41 | 0.425 | 248 | 13.16 | 0.45 | 0.647 | 42 | 12.12 | 0.97 | 0.319 |
|  | overweight | 21 | 14.27 | 1.5 |  | 17 | 13.98 | 1.69 |  | 4 | 15.54 | 3.67 |  |
| FAT (% total kcal) | normal | 290 | 36.00 | 1.00 | 0.947 | 248 | 36.00 | 1.00 | 0.827 | 42 | 36.00 | 2.00 | 0.758 |
|  | overweight | 21 | 36.00 | 2.00 |  | 17 | 36.00 | 2.00 |  | 4 | 34.00 | 4.00 |  |

**Supplementary Table S3 –** Correlations between the snack test results and the resting-state fMRI connectivity in PROTAIA: (A) total sample, (B) control group, (C) SGA group; and in MAVAN: (D) total sample, (E) control group, (F) SGA group. *p<0.05

| **(A)** PROTAIA  (total sample n=40) | | LeftOFC_LeftAmy | LeftOFC_RightAmy | LeftOFC_LeftDS | LeftOFC_RightDS | RightOFC_LeftAmy | RightOFC_RightAmy | RightOFC_LeftDS | RightOFC_RightDS | LeftOFC_LeftDLPFC | LeftOFC_RightDLPFC | RightOFC_LeftDLPFC | RightOFC_RightDLPFC |
| --- | --- | --- | --- | --- | --- | --- | --- | --- | --- | --- | --- | --- | --- |
| Amount spent (R$) | Pearson Correlation | .050 | -.265 | -.205 | -.297 | .086 | .100 | .071 | **.347^*^** | -.059 | -.140 | .093 | .159 |
|  | Sig. (2-tailed) | .760 | .098 | .204 | .062 | .600 | .538 | .664 | **.028** | .717 | .390 | .567 | .328 |
| Energy (kcal) | Pearson Correlation | .229 | -.102 | -.131 | -.285 | .171 | .179 | .015 | .204 | -.061 | -.274 | .184 | .183 |
|  | Sig. (2-tailed) | .155 | .530 | .420 | .075 | .291 | .270 | .927 | .206 | .710 | .087 | .257 | .259 |
| PTN (g) | Pearson Correlation | .279 | .057 | -.050 | -.092 | **.323^*^** | .307 | .034 | .191 | .079 | -.119 | .054 | .061 |
|  | Sig. (2-tailed) | .081 | .726 | .762 | .570 | **.042** | .054 | .835 | .238 | .630 | .463 | .740 | .707 |
| PTN (% total kcal) | Pearson Correlation | .148 | .283 | -.035 | .088 | .286 | **.324^*^** | -.007 | .031 | .074 | .072 | -.094 | -.103 |
|  | Sig. (2-tailed) | .362 | .077 | .829 | .589 | .073 | **.041** | .968 | .848 | .651 | .657 | .564 | .529 |
| CHO (g) | Pearson Correlation | .170 | -.147 | -.131 | **-.323^*^** | .095 | .126 | .052 | .223 | -.125 | **-.331^*^** | .164 | .266 |
|  | Sig. (2-tailed) | .294 | .367 | .421 | **.042** | .561 | .439 | .749 | .167 | .444 | **.037** | .310 | .098 |
| Sugar (g) | Pearson Correlation | .000 | -.202 | -.202 | **-.421^**^** | -.093 | -.003 | -.015 | .065 | -.247 | **-.322^*^** | .111 | .289 |
|  | Sig. (2-tailed) | .998 | .212 | .211 | **.007** | .569 | .987 | .928 | .689 | .125 | **.043** | .497 | .070 |
| Fiber (g) | Pearson Correlation | .278 | -.067 | -.161 | -.225 | **.332^*^** | .213 | .007 | .126 | -.063 | -.134 | .060 | .157 |
|  | Sig. (2-tailed) | .082 | .683 | .320 | .163 | **.036** | .186 | .966 | .439 | .698 | .410 | .714 | .335 |
| CHO (% total kcal) | Pearson Correlation | -.052 | -.155 | .068 | -.053 | -.157 | -.172 | .124 | .067 | -.107 | -.173 | -.048 | .174 |
|  | Sig. (2-tailed) | .750 | .339 | .677 | .746 | .333 | .290 | .444 | .682 | .512 | .286 | .766 | .284 |
| FAT (g) | Pearson Correlation | .210 | -.091 | -.140 | -.250 | .149 | .136 | -.048 | .121 | -.026 | -.190 | .221 | .073 |
|  | Sig. (2-tailed) | .193 | .578 | .388 | .120 | .360 | .401 | .770 | .458 | .872 | .240 | .170 | .654 |
| FAT (% total kcal) | Pearson Correlation | -.018 | .026 | -.081 | -.016 | .035 | .036 | -.147 | -.102 | .068 | .163 | .131 | -.153 |
|  | Sig. (2-tailed) | .912 | .871 | .617 | .924 | .832 | .827 | .365 | .533 | .676 | .314 | .420 | .346 |

| **(B)** PROTAIA  (controls n=33) | | LeftOFC_LeftAmy | LeftOFC_RightAmy | LeftOFC_LeftDS | LeftOFC_RightDS | RightOFC_LeftAmy | RightOFC_RightAmy | RightOFC_LeftDS | RightOFC_RightDS | LeftOFC_LeftDLPFC | LeftOFC_RightDLPFC | RightOFC_LeftDLPFC | RightOFC_RightDLPFC |
| --- | --- | --- | --- | --- | --- | --- | --- | --- | --- | --- | --- | --- | --- |
| Amount spent (R$) | Pearson Correlation | -.030 | -.099 | -.245 | -.343 | -.057 | .166 | .004 | .316 | -.228 | -.173 | .024 | .193 |
|  | Sig. (2-tailed) | .866 | .582 | .170 | .051 | .752 | .357 | .983 | .073 | .201 | .337 | .896 | .282 |
| Energy (kcal) | Pearson Correlation | .220 | .085 | -.097 | -.271 | .067 | .240 | -.046 | .179 | -.198 | -.323 | .129 | .236 |
|  | Sig. (2-tailed) | .219 | .637 | .593 | .127 | .713 | .179 | .801 | .319 | .269 | .067 | .475 | .186 |
| PTN (g) | Pearson Correlation | .260 | .158 | -.027 | -.066 | .260 | .335 | -.036 | .152 | -.033 | -.171 | -.052 | .069 |
|  | Sig. (2-tailed) | .144 | .379 | .881 | .714 | .145 | .056 | .843 | .398 | .855 | .342 | .774 | .703 |
| PTN (% total kcal) | Pearson Correlation | .189 | .217 | -.009 | .106 | .303 | .298 | -.054 | .048 | .064 | .024 | -.168 | -.107 |
|  | Sig. (2-tailed) | .293 | .226 | .959 | .556 | .087 | .092 | .764 | .792 | .724 | .895 | .350 | .555 |
| CHO (g) | Pearson Correlation | .158 | .053 | -.101 | -.330 | -.022 | .181 | -.004 | .173 | -.231 | **-.372*** | .155 | .335 |
|  | Sig. (2-tailed) | .379 | .771 | .577 | .061 | .905 | .313 | .984 | .336 | .195 | **.033** | .389 | .057 |
| Sugar (g) | Pearson Correlation | -.002 | -.028 | -.180 | **-.450**** | -.189 | .041 | -.030 | .018 | -.296 | -.309 | .161 | **.369*** |
|  | Sig. (2-tailed) | .992 | .876 | .317 | **.009** | .293 | .823 | .869 | .919 | .095 | .080 | .372 | **.034** |
| Fiber (g) | Pearson Correlation | .270 | .050 | -.098 | -.177 | .315 | .255 | -.080 | .128 | -.142 | -.152 | -.072 | .198 |
|  | Sig. (2-tailed) | .129 | .782 | .586 | .324 | .074 | .152 | .658 | .479 | .431 | .398 | .689 | .268 |
| CHO (% total kcal) | Pearson Correlation | -.097 | -.071 | .015 | -.125 | -.165 | -.144 | .181 | .006 | -.100 | -.167 | .031 | .193 |
|  | Sig. (2-tailed) | .591 | .693 | .933 | .489 | .359 | .424 | .314 | .975 | .579 | .354 | .862 | .283 |
| FAT (g) | Pearson Correlation | .200 | .058 | -.106 | -.222 | .061 | .191 | -.096 | .133 | -.177 | -.226 | .146 | .106 |
|  | Sig. (2-tailed) | .264 | .748 | .559 | .215 | .735 | .288 | .595 | .461 | .325 | .207 | .416 | .557 |
| FAT (% total kcal) | Pearson Correlation | .000 | -.044 | -.025 | .050 | .002 | -.003 | -.188 | -.045 | .044 | .173 | .075 | -.155 |
|  | Sig. (2-tailed) | .999 | .806 | .892 | .780 | .992 | .988 | .294 | .803 | .807 | .337 | .678 | .388 |

| **(C)** PROTAIA  (SGA n=7) | | LeftOFC_LeftAmy | LeftOFC_RightAmy | LeftOFC_LeftDS | LeftOFC_RightDS | RightOFC_LeftAmy | RightOFC_RightAmy | RightOFC_LeftDS | RightOFC_RightDS | LeftOFC_LeftDLPFC | LeftOFC_RightDLPFC | RightOFC_LeftDLPFC | RightOFC_RightDLPFC |
| --- | --- | --- | --- | --- | --- | --- | --- | --- | --- | --- | --- | --- | --- |
| Amount spent (R$) | Pearson Correlation | .164 | **-.944^**^** | -.240 | -.418 | -.035 | -.692 | -.270 | -.132 | -.070 | **-.769^*^** | -.020 | -.143 |
|  | Sig. (2-tailed) | .725 | **.001** | .604 | .350 | .941 | .085 | .558 | .778 | .881 | **.043** | .966 | .759 |
| Energy (kcal) | Pearson Correlation | .037 | **-.885^**^** | -.509 | -.628 | .117 | -.530 | -.282 | -.337 | -.060 | **-.757^*^** | .114 | -.296 |
|  | Sig. (2-tailed) | .938 | **.008** | .243 | .131 | .802 | .221 | .540 | .459 | .899 | **.049** | .807 | .520 |
| PTN (g) | Pearson Correlation | .067 | -.571 | -.489 | -.649 | .074 | -.357 | -.300 | -.552 | .120 | -.501 | .274 | -.251 |
|  | Sig. (2-tailed) | .886 | .181 | .266 | .114 | .875 | .431 | .514 | .199 | .798 | .252 | .552 | .587 |
| PTN (% total kcal) | Pearson Correlation | -.280 | .663 | -.254 | -.064 | .241 | .661 | .151 | -.227 | .046 | .487 | .210 | -.112 |
|  | Sig. (2-tailed) | .544 | .105 | .583 | .892 | .603 | .106 | .747 | .625 | .922 | .268 | .652 | .811 |
| CHO (g) | Pearson Correlation | .004 | **-.994^**^** | -.454 | -.521 | .178 | -.541 | -.150 | -.036 | -.240 | **-.788^*^** | -.055 | -.291 |
|  | Sig. (2-tailed) | .993 | **.000** | .307 | .230 | .702 | .210 | .748 | .939 | .605 | **.035** | .907 | .526 |
| Sugar (g) | Pearson Correlation | -.102 | **-.944^**^** | -.394 | -.368 | .207 | -.474 | -.107 | .128 | -.335 | **-.784^*^** | -.178 | -.307 |
|  | Sig. (2-tailed) | .828 | **.001** | .382 | .417 | .656 | .282 | .819 | .784 | .463 | **.037** | .702 | .503 |
| Fiber (g) | Pearson Correlation | .252 | -.578 | -.640 | -.610 | .307 | -.232 | .143 | -.213 | .015 | -.368 | .486 | -.230 |
|  | Sig. (2-tailed) | .586 | .174 | .121 | .146 | .504 | .616 | .759 | .646 | .975 | .416 | .268 | .620 |
| CHO (% total kcal) | Pearson Correlation | .222 | -.457 | .336 | .246 | -.178 | -.459 | .031 | .457 | -.145 | -.283 | -.287 | .163 |
|  | Sig. (2-tailed) | .633 | .303 | .461 | .596 | .702 | .301 | .947 | .302 | .756 | .539 | .533 | .728 |
| FAT (g) | Pearson Correlation | .067 | -.639 | -.478 | -.610 | .037 | -.444 | -.366 | -.569 | .131 | -.622 | .271 | -.253 |
|  | Sig. (2-tailed) | .887 | .123 | .277 | .146 | .938 | .319 | .420 | .182 | .779 | .136 | .557 | .584 |
| FAT (% total kcal) | Pearson Correlation | -.184 | .302 | -.397 | -.335 | .175 | .335 | -.094 | -.526 | .180 | .150 | .331 | -.218 |
|  | Sig. (2-tailed) | .692 | .510 | .378 | .463 | .707 | .463 | .841 | .225 | .699 | .748 | .469 | .638 |

| **(D)** MAVAN  (total sample n=42) | | LeftOFC_LeftAmy | LeftOFC_RightAmy | LeftOFC_LeftDS | LeftOFC_RightDS | RightOFC_LeftAmy | RightOFC_RightAmy | RightOFC_LeftDS | RightOFC_RightDS | LeftOFC_LeftDLPFC | LeftOFC_RightDLPFC | RightOFC_LeftDLPFC | RightOFC_RightDLPFC |
| --- | --- | --- | --- | --- | --- | --- | --- | --- | --- | --- | --- | --- | --- |
| Energy (kcal) | Pearson Correlation | -0.190 | -0.136 | -0.104 | -0.003 | -0.247 | -0.303 | 0.151 | 0.033 | -0.085 | 0.124 | -0.098 | -0.015 |
|  | Sig. (2-tailed) | 0.227 | 0.389 | 0.511 | 0.984 | 0.114 | 0.051 | 0.339 | 0.837 | 0.595 | 0.435 | 0.537 | 0.923 |
| PTN (g) | Pearson Correlation | -0.173 | -0.056 | -0.160 | -0.031 | -0.176 | -0.257 | 0.077 | -0.026 | -0.123 | -0.025 | -0.110 | -0.156 |
|  | Sig. (2-tailed) | 0.274 | 0.726 | 0.313 | 0.846 | 0.264 | 0.101 | 0.630 | 0.870 | 0.438 | 0.878 | 0.488 | 0.325 |
| PTN (% total kcal) | Pearson Correlation | -0.011 | 0.083 | -0.087 | -0.040 | 0.093 | 0.073 | -0.051 | -0.073 | -0.040 | -0.225 | -0.055 | **-0.345*** |
|  | Sig. (2-tailed) | 0.947 | 0.603 | 0.584 | 0.803 | 0.559 | 0.644 | 0.748 | 0.646 | 0.804 | 0.152 | 0.729 | **0.025** |
| CHO (g) | Pearson Correlation | -0.163 | -0.136 | -0.085 | -0.048 | -0.239 | **-0.317*** | 0.053 | -0.020 | -0.123 | 0.221 | -0.120 | 0.123 |
|  | Sig. (2-tailed) | 0.301 | 0.391 | 0.591 | 0.764 | 0.128 | **0.041** | 0.741 | 0.898 | 0.438 | 0.160 | 0.449 | 0.438 |
| Sugar (g) | Pearson Correlation | -0.052 | -0.200 | -0.015 | 0.018 | -0.277 | **-0.320*** | 0.156 | 0.250 | -0.146 | 0.063 | 0.028 | 0.110 |
|  | Sig. (2-tailed) | 0.744 | 0.204 | 0.923 | 0.907 | 0.075 | **0.039** | 0.323 | 0.110 | 0.355 | 0.691 | 0.860 | 0.488 |
| Fiber (g) | Pearson Correlation | -0.152 | 0.090 | 0.039 | -0.016 | 0.046 | 0.018 | 0.051 | -0.167 | -0.071 | **0.372*** | -0.037 | 0.216 |
|  | Sig. (2-tailed) | 0.336 | 0.569 | 0.805 | 0.922 | 0.774 | 0.911 | 0.747 | 0.290 | 0.656 | **0.015** | 0.816 | 0.169 |
| CHO (% total kcal) | Pearson Correlation | 0.064 | 0.049 | 0.049 | 0.025 | -0.021 | -0.094 | -0.091 | -0.026 | -0.055 | 0.178 | 0.007 | **0.318*** |
|  | Sig. (2-tailed) | 0.686 | 0.759 | 0.759 | 0.875 | 0.896 | 0.555 | 0.569 | 0.871 | 0.728 | 0.260 | 0.963 | **0.040** |
| FAT (g) | Pearson Correlation | -0.158 | -0.094 | -0.075 | 0.029 | -0.166 | -0.174 | 0.193 | 0.064 | -0.011 | 0.031 | -0.050 | -0.099 |
|  | Sig. (2-tailed) | 0.319 | 0.555 | 0.637 | 0.855 | 0.294 | 0.271 | 0.220 | 0.685 | 0.944 | 0.846 | 0.753 | 0.531 |
| FAT (% total kcal) | Pearson Correlation | -0.090 | -0.061 | -0.041 | -0.043 | 0.043 | 0.132 | 0.108 | 0.021 | 0.066 | -0.149 | -0.015 | -0.267 |
|  | Sig. (2-tailed) | 0.569 | 0.703 | 0.795 | 0.787 | 0.789 | 0.405 | 0.495 | 0.893 | 0.680 | 0.347 | 0.927 | 0.088 |

| **(E)** MAVAN  (controls n=29) | | LeftOFC_LeftAmy | LeftOFC_RightAmy | LeftOFC_LeftDS | LeftOFC_RightDS | RightOFC_LeftAmy | RightOFC_RightAmy | RightOFC_LeftDS | RightOFC_RightDS | LeftOFC_LeftDLPFC | LeftOFC_RightDLPFC | RightOFC_LeftDLPFC | RightOFC_RightDLPFC |
| --- | --- | --- | --- | --- | --- | --- | --- | --- | --- | --- | --- | --- | --- |
| Energy (kcal) | Pearson Correlation | -0.142 | -0.083 | -0.203 | -0.044 | -0.264 | -0.342 | 0.102 | -0.019 | -0.069 | 0.032 | -0.100 | -0.139 |
|  | Sig. (2-tailed) | 0.463 | 0.669 | 0.290 | 0.822 | 0.166 | 0.069 | 0.600 | 0.922 | 0.722 | 0.871 | 0.606 | 0.472 |
| PTN (g) | Pearson Correlation | -0.113 | -0.012 | -0.251 | -0.093 | -0.222 | -0.310 | -0.018 | -0.122 | -0.065 | -0.020 | -0.049 | -0.135 |
|  | Sig. (2-tailed) | 0.559 | 0.950 | 0.189 | 0.632 | 0.248 | 0.101 | 0.927 | 0.530 | 0.736 | 0.918 | 0.800 | 0.485 |
| PTN (% total kcal) | Pearson Correlation | -0.014 | 0.066 | -0.103 | -0.026 | 0.028 | 0.018 | -0.125 | -0.115 | 0.034 | -0.088 | 0.043 | -0.193 |
|  | Sig. (2-tailed) | 0.941 | 0.735 | 0.594 | 0.893 | 0.884 | 0.924 | 0.519 | 0.553 | 0.861 | 0.650 | 0.825 | 0.316 |
| CHO (g) | Pearson Correlation | -0.120 | -0.108 | -0.123 | -0.005 | -0.232 | -0.346 | 0.102 | 0.015 | -0.230 | -0.003 | -0.242 | -0.156 |
|  | Sig. (2-tailed) | 0.536 | 0.579 | 0.525 | 0.978 | 0.225 | 0.066 | 0.598 | 0.937 | 0.230 | 0.988 | 0.206 | 0.420 |
| Sugar (g) | Pearson Correlation | -0.023 | -0.113 | -0.066 | -0.043 | -0.199 | -0.257 | 0.203 | 0.224 | -0.251 | -0.075 | -0.086 | 0.007 |
|  | Sig. (2-tailed) | 0.906 | 0.561 | 0.735 | 0.824 | 0.301 | 0.178 | 0.291 | 0.243 | 0.189 | 0.700 | 0.656 | 0.973 |
| Fiber (g) | Pearson Correlation | 0.127 | 0.118 | 0.221 | 0.246 | 0.061 | 0.084 | 0.191 | -0.003 | -0.074 | 0.410 | 0.044 | 0.122 |
|  | Sig. (2-tailed) | 0.510 | 0.543 | 0.250 | 0.198 | 0.754 | 0.664 | 0.322 | 0.988 | 0.704 | 0.027 | 0.819 | 0.527 |
| CHO (% total kcal) | Pearson Correlation | 0.134 | 0.100 | 0.233 | 0.138 | 0.111 | 0.028 | 0.062 | 0.078 | -0.144 | 0.081 | -0.057 | 0.235 |
|  | Sig. (2-tailed) | 0.487 | 0.605 | 0.225 | 0.477 | 0.567 | 0.887 | 0.748 | 0.689 | 0.457 | 0.676 | 0.769 | 0.220 |
| FAT (g) | Pearson Correlation | -0.136 | -0.065 | -0.204 | -0.057 | -0.233 | -0.256 | 0.106 | -0.019 | 0.062 | 0.060 | 0.004 | -0.096 |
|  | Sig. (2-tailed) | 0.482 | 0.739 | 0.289 | 0.771 | 0.224 | 0.180 | 0.585 | 0.921 | 0.748 | 0.756 | 0.983 | 0.619 |
| FAT (% total kcal) | Pearson Correlation | -0.165 | -0.132 | -0.250 | -0.166 | -0.107 | -0.003 | -0.050 | -0.068 | 0.167 | -0.071 | 0.053 | -0.186 |
|  | Sig. (2-tailed) | 0.394 | 0.495 | 0.191 | 0.391 | 0.580 | 0.987 | 0.795 | 0.725 | 0.386 | 0.716 | 0.786 | 0.335 |

| **(F)** MAVAN  (SGA n=13) | | LeftOFC_LeftAmy | LeftOFC_RightAmy | LeftOFC_LeftDS | LeftOFC_RightDS | RightOFC_LeftAmy | RightOFC_RightAmy | RightOFC_LeftDS | RightOFC_RightDS | LeftOFC_LeftDLPFC | LeftOFC_RightDLPFC | RightOFC_LeftDLPFC | RightOFC_RightDLPFC |
| --- | --- | --- | --- | --- | --- | --- | --- | --- | --- | --- | --- | --- | --- |
| Energy (kcal) | Pearson Correlation | -0.322 | -0.250 | 0.187 | 0.106 | -0.160 | -0.138 | 0.309 | 0.197 | -0.039 | 0.469 | -0.021 | 0.347 |
|  | Sig. (2-tailed) | 0.283 | 0.411 | 0.540 | 0.731 | 0.602 | 0.652 | 0.305 | 0.520 | 0.900 | 0.106 | 0.946 | 0.246 |
| PTN (g) | Pearson Correlation | -0.322 | -0.073 | 0.134 | 0.146 | 0.067 | -0.007 | 0.376 | 0.297 | -0.192 | 0.087 | -0.216 | -0.224 |
|  | Sig. (2-tailed) | 0.283 | 0.813 | 0.661 | 0.635 | 0.828 | 0.981 | 0.205 | 0.325 | 0.529 | 0.778 | 0.479 | 0.461 |
| PTN (% total kcal) | Pearson Correlation | -0.005 | 0.121 | -0.062 | -0.061 | 0.242 | 0.226 | 0.067 | 0.000 | -0.195 | -0.428 | -0.248 | -0.590 |
|  | Sig. (2-tailed) | 0.986 | 0.694 | 0.840 | 0.844 | 0.425 | 0.457 | 0.829 | 0.999 | 0.523 | 0.145 | 0.413 | 0.034 |
| CHO (g) | Pearson Correlation | -0.319 | -0.246 | -0.051 | -0.102 | -0.292 | -0.285 | -0.006 | -0.073 | -0.047 | 0.424 | -0.001 | 0.470 |
|  | Sig. (2-tailed) | 0.288 | 0.418 | 0.868 | 0.740 | 0.333 | 0.345 | 0.984 | 0.813 | 0.879 | 0.149 | 0.997 | 0.105 |
| Sugar (g) | Pearson Correlation | -0.189 | -0.438 | 0.040 | 0.102 | -0.490 | -0.500 | 0.097 | 0.295 | -0.084 | 0.166 | 0.158 | 0.242 |
|  | Sig. (2-tailed) | 0.537 | 0.135 | 0.897 | 0.741 | 0.089 | 0.082 | 0.752 | 0.328 | 0.786 | 0.589 | 0.606 | 0.426 |
| Fiber (g) | Pearson Correlation | -0.595 | 0.123 | -0.178 | -0.323 | 0.048 | -0.128 | -0.131 | -0.398 | 0.002 | 0.430 | -0.100 | 0.353 |
|  | Sig. (2-tailed) | 0.032 | 0.688 | 0.561 | 0.281 | 0.877 | 0.677 | 0.669 | 0.178 | 0.994 | 0.142 | 0.745 | 0.237 |
| CHO (% total kcal) | Pearson Correlation | -0.140 | -0.097 | -0.261 | -0.140 | -0.341 | -0.413 | -0.321 | -0.203 | 0.014 | 0.264 | 0.060 | 0.434 |
|  | Sig. (2-tailed) | 0.649 | 0.753 | 0.389 | 0.649 | 0.254 | 0.161 | 0.285 | 0.507 | 0.964 | 0.384 | 0.847 | 0.138 |
| FAT (g) | Pearson Correlation | -0.067 | 0.002 | 0.474 | 0.361 | 0.268 | 0.293 | 0.592 | 0.446 | 0.023 | 0.223 | -0.030 | -0.084 |
|  | Sig. (2-tailed) | 0.829 | 0.995 | 0.101 | 0.226 | 0.376 | 0.331 | 0.033 | 0.126 | 0.941 | 0.464 | 0.922 | 0.785 |
| FAT (% total kcal) | Pearson Correlation | 0.133 | 0.132 | 0.312 | 0.136 | 0.411 | 0.489 | 0.347 | 0.175 | -0.008 | -0.197 | -0.059 | -0.376 |
|  | Sig. (2-tailed) | 0.665 | 0.668 | 0.299 | 0.657 | 0.163 | 0.090 | 0.246 | 0.568 | 0.978 | 0.519 | 0.848 | 0.206 |

**Supplementary Table S4 –** Correlations between the energy and macronutrients’ consumption per kg and the resting-state fMRI connectivity in PROTAIA: (A) total sample, (B) control group, (C) SGA group; and in MAVAN: (D) total sample, (E) control group, (F) SGA group. *p<0.05

| **(A)** PROTAIA  (total sample n=40) | | LeftOFC_LeftAmy | LeftOFC_RightAmy | LeftOFC_LeftDS | LeftOFC_RightDS | RightOFC_LeftAmy | RightOFC_RightAmy | RightOFC_LeftDS | RightOFC_RightDS | LeftOFC_LeftDLPFC | LeftOFC_RightDLPFC | RightOFC_LeftDLPFC | RightOFC_RightDLPFC |
| --- | --- | --- | --- | --- | --- | --- | --- | --- | --- | --- | --- | --- | --- |
| Energy (kcal/kg) | Pearson Correlation | .217 | -.095 | -.100 | -.268 | .172 | .111 | .070 | .160 | -.190 | -.289 | .100 | .187 |
|  | Sig. (2-tailed) | .179 | .560 | .538 | .094 | .288 | .497 | .669 | .324 | .241 | .070 | .539 | .249 |
| PTN (g/kg) | Pearson Correlation | .251 | .065 | -.028 | -.070 | **.332^*^** | .270 | .063 | .167 | .006 | -.111 | .017 | .094 |
|  | Sig. (2-tailed) | .117 | .690 | .865 | .669 | **.036** | .092 | .701 | .302 | .968 | .495 | .919 | .564 |
| CHO (g/kg) | Pearson Correlation | .168 | -.137 | -.097 | -.302 | .114 | .066 | .114 | .185 | -.254 | **-.342^*^** | .081 | .256 |
|  | Sig. (2-tailed) | .300 | .400 | .551 | .058 | .482 | .685 | .485 | .252 | .114 | **.031** | .620 | .111 |
| Sugars (g/kg) | Pearson Correlation | .024 | -.185 | -.164 | **-.393^*^** | -.047 | -.028 | .044 | .049 | **-.335^*^** | **-.327^*^** | .032 | .255 |
|  | Sig. (2-tailed) | .885 | .253 | .311 | **.012** | .773 | .866 | .789 | .765 | **.034** | **.040** | .845 | .113 |
| FAT (g/kg) | Pearson Correlation | .202 | -.084 | -.116 | -.244 | .133 | .074 | -.005 | .077 | -.138 | -.216 | .140 | .082 |
|  | Sig. (2-tailed) | .211 | .607 | .475 | .129 | .413 | .652 | .977 | .637 | .397 | .180 | .388 | .615 |

| **(B)** PROTAIA  (controls n=33) | | LeftOFC_LeftAmy | LeftOFC_RightAmy | LeftOFC_LeftDS | LeftOFC_RightDS | RightOFC_LeftAmy | RightOFC_RightAmy | RightOFC_LeftDS | RightOFC_RightDS | LeftOFC_LeftDLPFC | LeftOFC_RightDLPFC | RightOFC_LeftDLPFC | RightOFC_RightDLPFC |
| --- | --- | --- | --- | --- | --- | --- | --- | --- | --- | --- | --- | --- | --- |
| Energy (kcal/kg) | Pearson Correlation | .256 | .128 | -.038 | -.229 | .130 | .186 | .123 | .212 | -.318 | -.291 | .080 | .277 |
|  | Sig. (2-tailed) | .150 | .477 | .834 | .200 | .472 | .300 | .494 | .235 | .071 | .100 | .657 | .119 |
| PTN (g/kg) | Pearson Correlation | .253 | .192 | .009 | -.024 | .309 | .314 | .063 | .182 | -.097 | -.124 | -.071 | .120 |
|  | Sig. (2-tailed) | .156 | .283 | .961 | .895 | .081 | .075 | .727 | .311 | .591 | .492 | .693 | .505 |
| CHO (g/kg) | Pearson Correlation | .207 | .098 | -.033 | -.278 | .051 | .130 | .155 | .201 | **-.350^*^** | **-.347^*^** | .106 | **.366^*^** |
|  | Sig. (2-tailed) | .247 | .589 | .856 | .117 | .778 | .470 | .389 | .261 | **.046** | **.048** | .556 | **.036** |
| Sugars (g/kg) | Pearson Correlation | .062 | .014 | -.114 | **-.398^*^** | -.107 | .019 | .092 | .046 | **-.375^*^** | -.295 | .106 | **.366^*^** |
|  | Sig. (2-tailed) | .730 | .940 | .529 | **.022** | .553 | .915 | .609 | .799 | **.032** | .096 | .556 | **.036** |
| FAT (g/kg) | Pearson Correlation | .235 | .094 | -.065 | -.201 | .108 | .145 | .066 | .169 | -.286 | -.206 | .094 | .141 |
|  | Sig. (2-tailed) | .187 | .602 | .718 | .262 | .548 | .421 | .716 | .346 | .106 | .250 | .603 | .434 |

| **(C)** PROTAIA  (SGA n=7) | | LeftOFC_LeftAmy | LeftOFC_RightAmy | LeftOFC_LeftDS | LeftOFC_RightDS | RightOFC_LeftAmy | RightOFC_RightAmy | RightOFC_LeftDS | RightOFC_RightDS | LeftOFC_LeftDLPFC | LeftOFC_RightDLPFC | RightOFC_LeftDLPFC | RightOFC_RightDLPFC |
| --- | --- | --- | --- | --- | --- | --- | --- | --- | --- | --- | --- | --- | --- |
| Energy (kcal/kg) | Pearson Correlation | -.103 | **-.872^*^** | -.489 | -.586 | .114 | -.514 | -.381 | -.358 | -.067 | -.808^*^ | .015 | -.389 |
|  | Sig. (2-tailed) | .825 | **.010** | .265 | .167 | .807 | .237 | .399 | .430 | .886 | .028 | .975 | .388 |
| PTN (g/kg) | Pearson Correlation | -.049 | -.623 | -.449 | -.591 | .018 | -.433 | -.466 | -.608 | .129 | -.652 | .175 | -.319 |
|  | Sig. (2-tailed) | .917 | .135 | .312 | .162 | .970 | .332 | .292 | .148 | .783 | .112 | .708 | .486 |
| CHO (g/kg) | Pearson Correlation | -.142 | **-.972^**^** | -.481 | -.529 | .221 | -.483 | -.211 | -.060 | -.252 | **-.799^*^** | -.130 | -.418 |
|  | Sig. (2-tailed) | .761 | **.000** | .274 | .222 | .634 | .272 | .650 | .898 | .585 | **.031** | .781 | .351 |
| Sugars (g/kg) | Pearson Correlation | -.212 | **-.939^**^** | -.449 | -.427 | .270 | -.409 | -.126 | .110 | -.360 | **-.766^*^** | -.230 | -.417 |
|  | Sig. (2-tailed) | .649 | **.002** | .313 | .339 | .558 | .362 | .788 | .814 | .427 | **.045** | .619 | .352 |
| FAT (g/kg) | Pearson Correlation | -.041 | -.659 | -.431 | -.552 | -.011 | -.487 | -.499 | -.606 | .133 | -.722 | .171 | -.305 |
|  | Sig. (2-tailed) | .931 | .107 | .334 | .199 | .981 | .267 | .254 | .149 | .776 | .067 | .714 | .506 |

| **(D)** MAVAN  (total sample n=42) | | LeftOFC_LeftAmy | LeftOFC_RightAmy | LeftOFC_LeftDS | LeftOFC_RightDS | RightOFC_LeftAmy | RightOFC_RightAmy | RightOFC_LeftDS | RightOFC_RightDS | LeftOFC_LeftDLPFC | LeftOFC_RightDLPFC | RightOFC_LeftDLPFC | RightOFC_RightDLPFC |
| --- | --- | --- | --- | --- | --- | --- | --- | --- | --- | --- | --- | --- | --- |
| Energy (kcal/kg) | Pearson Correlation | -0.190 | -0.014 | 0.037 | 0.050 | -0.106 | -0.158 | 0.230 | 0.091 | 0.046 | 0.297 | -0.032 | 0.019 |
|  | Sig. (2-tailed) | 0.234 | 0.932 | 0.816 | 0.757 | 0.509 | 0.324 | 0.148 | 0.572 | 0.776 | 0.059 | 0.843 | 0.906 |
| PTN (g/kg) | Pearson Correlation | -0.166 | 0.051 | -0.045 | 0.036 | -0.042 | -0.110 | 0.150 | 0.034 | -0.041 | 0.110 | -0.096 | -0.179 |
|  | Sig. (2-tailed) | 0.299 | 0.754 | 0.782 | 0.825 | 0.794 | 0.493 | 0.348 | 0.835 | 0.800 | 0.494 | 0.549 | 0.264 |
| CHO (g/kg) | Pearson Correlation | -0.181 | -0.006 | 0.041 | -0.028 | -0.103 | -0.197 | 0.107 | 0.014 | 0.012 | **0.385*** | -0.019 | 0.196 |
|  | Sig. (2-tailed) | 0.257 | 0.969 | 0.800 | 0.862 | 0.524 | 0.217 | 0.507 | 0.932 | 0.938 | **0.013** | 0.904 | 0.220 |
| Sugars (g/kg) | Pearson Correlation | -0.057 | -0.090 | 0.127 | 0.070 | -0.172 | -0.219 | 0.224 | 0.314 | -0.008 | 0.229 | 0.131 | 0.171 |
|  | Sig. (2-tailed) | 0.725 | 0.576 | 0.429 | 0.666 | 0.284 | 0.169 | 0.158 | 0.046 | 0.961 | 0.150 | 0.413 | 0.285 |
| FAT (g/kg) | Pearson Correlation | -0.136 | -0.016 | 0.028 | 0.087 | -0.065 | -0.048 | 0.260 | 0.119 | 0.077 | 0.133 | -0.028 | -0.114 |
|  | Sig. (2-tailed) | 0.397 | 0.921 | 0.861 | 0.587 | 0.686 | 0.764 | 0.100 | 0.459 | 0.632 | 0.406 | 0.862 | 0.477 |

| **(E)** MAVAN  (controls n=29) | | LeftOFC_LeftAmy | LeftOFC_RightAmy | LeftOFC_LeftDS | LeftOFC_RightDS | RightOFC_LeftAmy | RightOFC_RightAmy | RightOFC_LeftDS | RightOFC_RightDS | LeftOFC_LeftDLPFC | LeftOFC_RightDLPFC | RightOFC_LeftDLPFC | RightOFC_RightDLPFC |
| --- | --- | --- | --- | --- | --- | --- | --- | --- | --- | --- | --- | --- | --- |
| Energy (kcal/kg) | Pearson Correlation | -0.115 | -0.033 | -0.061 | 0.060 | -0.176 | -0.205 | 0.203 | 0.065 | 0.067 | 0.158 | -0.042 | -0.165 |
|  | Sig. (2-tailed) | 0.551 | 0.863 | 0.753 | 0.756 | 0.360 | 0.286 | 0.292 | 0.738 | 0.730 | 0.413 | 0.829 | 0.392 |
| PTN (g/kg) | Pearson Correlation | -0.099 | 0.023 | -0.124 | 0.007 | -0.146 | -0.185 | 0.083 | -0.047 | 0.060 | 0.099 | 0.000 | -0.183 |
|  | Sig. (2-tailed) | 0.610 | 0.905 | 0.521 | 0.972 | 0.451 | 0.336 | 0.668 | 0.809 | 0.757 | 0.611 | 1.000 | 0.342 |
| CHO (g/kg) | Pearson Correlation | -0.089 | -0.046 | 0.019 | 0.099 | -0.132 | -0.208 | 0.190 | 0.095 | -0.105 | 0.123 | -0.176 | -0.154 |
|  | Sig. (2-tailed) | 0.645 | 0.813 | 0.921 | 0.610 | 0.495 | 0.280 | 0.325 | 0.623 | 0.589 | 0.527 | 0.361 | 0.424 |
| Sugars (g/kg) | Pearson Correlation | 0.024 | -0.044 | 0.080 | 0.055 | -0.114 | -0.131 | 0.290 | 0.304 | -0.111 | 0.047 | -0.005 | 0.012 |
|  | Sig. (2-tailed) | 0.901 | 0.819 | 0.680 | 0.779 | 0.556 | 0.500 | 0.128 | 0.109 | 0.565 | 0.808 | 0.978 | 0.949 |
| FAT (g/kg) | Pearson Correlation | -0.114 | -0.031 | -0.092 | 0.020 | -0.169 | -0.144 | 0.187 | 0.047 | 0.178 | 0.156 | 0.047 | -0.128 |
|  | Sig. (2-tailed) | 0.555 | 0.875 | 0.636 | 0.916 | 0.382 | 0.457 | 0.330 | 0.810 | 0.356 | 0.419 | 0.809 | 0.509 |

| **(F)** MAVAN  (SGA n=13) | | LeftOFC_LeftAmy | LeftOFC_RightAmy | LeftOFC_LeftDS | LeftOFC_RightDS | RightOFC_LeftAmy | RightOFC_RightAmy | RightOFC_LeftDS | RightOFC_RightDS | LeftOFC_LeftDLPFC | LeftOFC_RightDLPFC | RightOFC_LeftDLPFC | RightOFC_RightDLPFC |
| --- | --- | --- | --- | --- | --- | --- | --- | --- | --- | --- | --- | --- | --- |
| Energy (kcal/kg) | Pearson Correlation | -0.514 | 0.012 | 0.279 | 0.029 | 0.112 | 0.026 | 0.321 | 0.163 | -0.035 | 0.615 | -0.024 | 0.449 |
|  | Sig. (2-tailed) | 0.088 | 0.971 | 0.379 | 0.928 | 0.730 | 0.936 | 0.309 | 0.614 | 0.915 | 0.033 | 0.941 | 0.143 |
| PTN (g/kg) | Pearson Correlation | -0.379 | 0.192 | 0.168 | 0.115 | 0.341 | 0.196 | 0.325 | 0.250 | -0.311 | 0.214 | -0.377 | -0.181 |
|  | Sig. (2-tailed) | 0.224 | 0.550 | 0.603 | 0.722 | 0.278 | 0.541 | 0.303 | 0.433 | 0.325 | 0.504 | 0.227 | 0.574 |
| CHO (g/kg) | Pearson Correlation | -0.561 | -0.085 | 0.051 | -0.186 | -0.124 | -0.213 | 0.049 | -0.085 | 0.034 | 0.557 | 0.104 | **0.602*** |
|  | Sig. (2-tailed) | 0.058 | 0.792 | 0.874 | 0.562 | 0.701 | 0.506 | 0.880 | 0.794 | 0.917 | 0.060 | 0.747 | **0.038** |
| Sugars (g/kg) | Pearson Correlation | -0.423 | -0.316 | 0.183 | 0.074 | -0.365 | -0.455 | 0.180 | 0.348 | 0.007 | 0.344 | 0.283 | 0.387 |
|  | Sig. (2-tailed) | 0.170 | 0.317 | 0.568 | 0.819 | 0.244 | 0.138 | 0.576 | 0.267 | 0.983 | 0.274 | 0.373 | 0.214 |
| FAT (g/kg) | Pearson Correlation | -0.062 | 0.212 | 0.489 | 0.355 | 0.488 | 0.470 | 0.547 | 0.409 | -0.101 | 0.313 | -0.205 | -0.081 |
|  | Sig. (2-tailed) | 0.849 | 0.509 | 0.107 | 0.257 | 0.107 | 0.123 | 0.066 | 0.187 | 0.756 | 0.322 | 0.522 | 0.804 |
